# AI4Green4Students: Promoting Sustainable Chemistry in Undergraduate Laboratories with an Electronic Lab Notebook

**DOI:** 10.1021/acs.jchemed.4c01393

**Published:** 2025-06-05

**Authors:** Peace C. Nwafor, Shason Gurung, Philip van Krimpen, Lenka Schnaubert, Katherine Jolley, Samantha Pearman-Kanza, Cerys Willoughby, Jonathan D. Hirst

**Affiliations:** † School of Chemistry, 6123 University of Nottingham, University Park, Nottingham NG7 2RD, United Kingdom; ‡ Digital Research Service, 6123 University of Nottingham, University Park Nottingham NG7 2RD, United Kingdom; § Learning Sciences Research Institute, School of Education, 6123 University of Nottingham, Dearing Building (C85), Jubilee Campus, Nottingham NG8 1BB, United Kingdom; ∥ School of Chemistry and Chemical Engineering, 7423 University of Southampton, University Road Southampton SO17 1BJ, United Kingdom

**Keywords:** Green Metrics, ELN, Sustainable Chemistry, Undergraduate Laboratory, Collaborative Learning

## Abstract

AI4Green is an open-source, machine-learning-powered electronic laboratory notebook (ELN) developed to help chemists mitigate environmental impacts, particularly within the pharmaceutical sector. This study presents AI4Green4Students, a pedagogically adapted version designed to foster sustainable laboratory practices. The AI4Green4Students ELN includes features for sustainability assessment, data documentation, and analysis, aiming to equip students with skills for modern, sustainable laboratory practices. User feedback, gathered through questionnaires and interviews, informed the initial development and subsequent refinements of the application. Implementation in a Year 3 undergraduate chemistry lab project indicated improved adherence to sustainable practices, suggesting that the ELN supports the integration of sustainable chemistry into laboratory teaching. Future efforts will expand AI4Green4Students to include additional experiments to broaden its applicability across undergraduate chemistry courses, as well as integrate machine learning tools to enhance data analysis capabilities.

## Graphical abstract

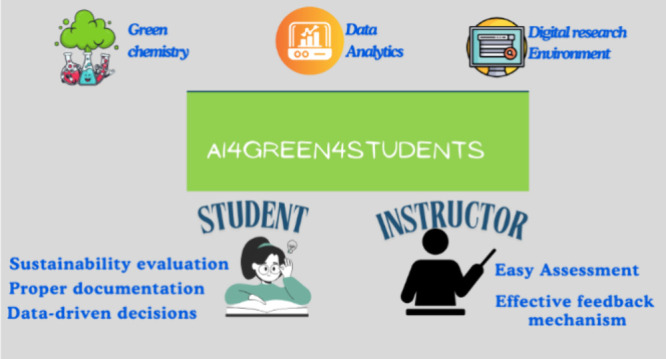

## Introduction

### Sustainable Chemistry

Sustainable chemistry promotes resource efficiency and reduces energy use, aligning with the United Nations (UN) Sustainable Development Goals (SDGs).^1^ It supports goals like ‘Good Health and Well-being’, ‘Responsible Consumption and Production’, and ‘Climate Action’ by fostering sustainable production and lowering emissions. This approach integrates environmental, economic, and social factors, utilizing chemical data intelligence to identify sustainable pathways and promote a circular chemical economy.^2^ Universities play a crucial role in achieving the SDGs.^3^ To empower students as agents of sustainable change, teaching methods and chemistry curricula must focus on green and sustainable chemistry.^4^ ELNs enable the practical application of sustainable principles in laboratory settings by improving documentation, promoting data sharing, and enhancing engagement with sustainability goals.^5^

### Green Chemistry and Its Integration into Education

Green chemistry, integral to promoting sustainability since the 19th century, focuses on pollution control by using renewable energy sources and replacing polluting technologies with safer alternatives. Anastas and Werner’s 12 principles of green chemistry^6^ guide the design of chemicals and products that minimize hazardous substances and waste. Scientists and engineers use green metrics^7^ (Table 1) to quantify how ‘green’ their chemical processes are.

**Table 1:** Selected Metrics Used to Evaluate Green Chemistry

*Green Metrics*	*Definition*	*Expression*
E-factor	Amount of waste generated per unit of product	Total waste (kg)/ mass of products
Process mass Intensity (PMI)	Total mass used in a process or process step divided by the mass of the product	Total mass in a process (incl. H_2_O)/Mass of product
Reaction mass efficiency (RME)	Mass efficiency of chemical process	(Mass of isolated product/total mass of reactants used in reaction) × 100

Since the publication of the textbook *Green Chemistry: Theory and Practice,* ^6^ green chemistry has increasingly been integrated into undergraduate education,^8^ aligning with the fourth UNSDG on education for sustainable development by 2030.^9^ There are two primary approaches to integrating green chemistry into curricula: embedding it within existing courses or offering stand-alone modules.^10^ Embedding green and sustainable chemistry into courses, particularly organic chemistry, allows students to apply sustainability principles directly to decision-making processes similar to those used in industry.^10,11^

Case studies^12^ and cooperative lab-based experiments^13^ have been effective in implementing green chemistry principles, alongside game-based activities^14^ and systems thinking approaches.^15^ However, challenges persist in fully integrating sustainability into chemistry curricula,^16–19^ including a lack of standardization, limited resources, and resistance to change.^20^ Traditional organic synthesis, focusing on petroleum-derived hydrocarbons, remains dominant due to its familiarity; yield and purity are emphasized over sustainability. This approach can confine students to small-scale, controlled laboratory conditions, emphasizing fundamental relationships rather than decision-making and practical application.^21^ Furthermore, traditional courses often rely heavily on toxic solvents and metal catalysts, hazardous to humans and the environment.^22,23^

To achieve full sustainability in chemistry education, life cycle analysis and toxicity testing should complement green chemistry principles, with digital tools driving sustainable practices.^13^ Digital tools, when employed in a technically meaningful and didactically reflective manner, can enhance the value of science education.^24^ For instance, our ELN integrates sustainability tools, including green metrics, solvent selection, and substitution frameworks. Interactive and engaging features can help sustain students’ interest and enhance the teaching-learning process.^24^ The adoption of these technologies can accelerate the learning and application of sustainable practices, while equipping students with industry-relevant skills.^25^

### Electronic Laboratory Notebooks in Teaching Laboratories

ELNs can enhance teaching laboratories by improving experimental documentation, promoting sustainability,^26^ and managing large data sets that paper laboratory notebooks cannot handle.^27^ Despite these benefits, their adoption in academia has been limited, due to factors such as resource constraints, unstandardized regulations, data security concerns, and resistance to change,^28,29^ as well as software interoperability.^30,31^ Costs of software subscription and hardware, coupled with compatibility challenges in multidisciplinary laboratories, also present barriers.^32^

ELNs facilitate data sharing, improve documentation practices, and enhance the reliability and reproducibility of scientific data.^28^ They streamline workflow management, enforce best practices, and incorporate tools such as risk-assessment templates to improve safety and compliance.^26,33^ ELNs support data-driven research and machine learning,^34^ helping students develop research data management skills, and promoting collaboration.^35^ They also enhance computer-assisted learning,^36^ goal focus and research,^37^ and can provide real-time monitoring and feedback to reduce errors in laboratory experiments.^38^

Recent studies have demonstrated successful ELN implementation in teaching laboratories, e.g., LabArchives in bioprocess engineering^26^ and Chemotion in inorganic chemistry.^35^ However, many ELNs do not focus on sustainable chemistry. AI4Green4Students, a cloud-based free ELN introduced in this study, addresses this gap by facilitating the learning and application of sustainable chemistry principles. It incorporates green metrics evaluation, risk-assessment templates, and feedback systems that align with sustainability goals. It provides a digital research environment (DRE) to streamline data management and ensure adherence to FAIR principles (Findability, Accessibility, Interoperability, and Reusability).^30^ AI4Green4Students also improves data recording, sharing, and submission processes while enabling real-time monitoring and feedback for instructors.

### The Role of Evaluation in Software Development

Evaluation plays a crucial role in software development.^39^ It can be formative, identifying weaknesses during development, or summative, verifying compliance with standards at the final stage.^40^ Usability testing examines how users interact with technology. According to the ISO 9421 framework, usability is defined by effectiveness, efficiency, and satisfaction.^39^ Iterative testing helps developers refine interfaces, enhancing user experience and increasing the likelihood of adoption. The Unified Theory of Acceptance and Use of Technology (UTAUT2) highlights factors influencing technology adoption, including performance expectancy, effort expectancy, social influence, facilitating conditions, hedonic motivation, price value, and habit.^41^

Questionnaires offer a structured, scalable way to gather both quantitative and qualitative feedback.^41^ They help evaluate usability aspects such as ease of use and satisfaction.^40^ In this study, questionnaires and interviews were used to gather feedback on the AI4Green4Students ELN. Insights informed its development, particularly its integration into a third-year chemistry lab course. The study’s findings, including user feedback and its impact on student learning, are presented, along with future directions for digital tools in sustainable chemistry education.

## Methods

### Study Design

This proof-of-concept study demonstrates the feasibility of integrating sustainable chemistry practices using an ELN. It employs a mixed-methods approach, with both quantitative and qualitative analyses to evaluate the usability of the AI4Green4Students ELN. The research consists of the development phase of the ELN, followed by usability testing involving students engaged in a four-week mini-laboratory project focused on Pd-catalyzed Suzuki cross-coupling reactions. Twelve third-year chemistry students (six males and six females), who are currently enrolled in an integrated master’s Chemistry program at the University of Nottingham, participated in the study. The objective was to assess how AI4Green4Students enables the application of sustainable chemistry principles, identify usability issues early and explore key features for documenting organic synthesis reactions.

Of the 12 participants, six were the control group and six the test group. The former used only OneNote Notebook for documentation while the latter utilized AI4Green4Students. Both the control and the test groups were given resources on green and sustainable chemistry (see sustainability cheatsheet in the Supporting Information) to bridge knowledge gaps on the topic before conducting lab experiments. Of the six participants in the test group, four had completed an optional sustainable chemistry module in their first and second years. All students had prior experience using the generic OneNote Notebook to plan and record lab experiments, but none had previously worked with an ELN. There were no specific selection criteria for participation. Students assigned to an instructor collaborating on this project were recruited following approval by the Ethics Committee, a full explanation of the study was provided, and written informed consent was obtained from all participants. Prior to the commencement of the study, the survey questions, along with the participant information form, which outlined the study’s purpose, the procedures involved, assurances of confidentiality, the voluntary nature of participation, and a request for informed consent, were submitted for ethical review. Our departmental ethics officer evaluated and ultimately approved the submitted documents.

### Development of AI4Green4Students

#### Overview

AI4Green4Students^42^ is a pedagogical version of AI4Green,^43^ an open-source, web-based application providing the essential functions of an ELN for research-level synthetic chemistry, promoting green and sustainable practices. AI4Green4Students ELN is designed to simplify sustainable chemistry in undergraduate teaching laboratories. Interviews and surveys conducted with students and instructors revealed challenges, especially with drawing reaction schemes and creating tables, using the current OneNote software. The AI4Green4Students ELN was developed to enhance the documentation process and address these limitations.

Initial wireframes^44^ (Figures S1–S3) and prototype designs were developed using Figma, with iterative improvements implemented through an agile methodology informed by continuous evaluation and user feedback. AI4Green4Students utilizes a C# and ASP.NET back end, integrated with Entity Framework for database management, and a React-driven front end^45^ for responsive and interactive features^46–48^ (see Figure 1).

**Figure 1:**
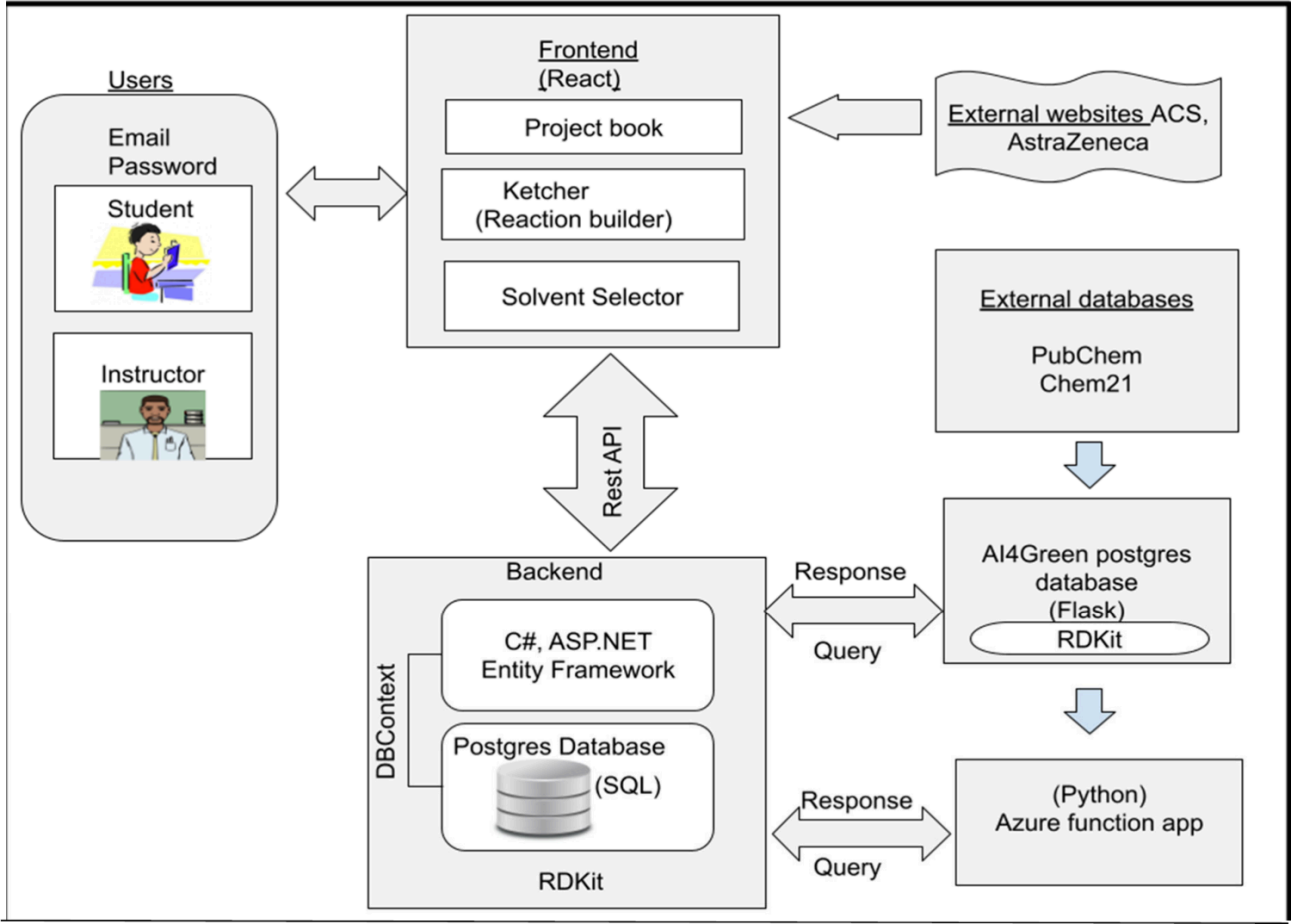
Architecture of AI4Green4Students, showing the databases, programming languages, and user interaction.

The application is cloud-hosted and utilizes Docker^49^ for containerized deployment. It integrates chemistry libraries like the Chemistry Development Kit (CDK) for molecular manipulation and Reaction Discovery Kit (RDKit)^50^ for cheminformatics, including reaction modeling and substructure searching. AI4Green4Students supports SMILES notation^51^ for chemical data input and connects to the PubChem database^52^ for consistent handling of chemical data. Data management is powered by a Postgres relational database,^53^ which organizes users, projects, solvents, lab notes, and associated data. Real-time calculations of green metrics derived from CHEM21 data^54^ are enabled, and reports can be generated, exported in Word and submitted via Moodle.

### Pedagogical Tools

#### Sustainability Assessment

Green metrics offer a quantitative approach to evaluating chemical products with respect to overall safety, environmental impact, and health implications.^7^ In the AI4Green4Students platform, students utilize the green metrics calculator to assess the greenness of chemical reactions by calculating parameters such as RME, PMI, E-factor, and Waste Intensity. The ELN incorporates supplementary tools to enhance sustainability assessment. The sustainable elements table identifies metals endangered due to extensive usage and links to information on their exploitation and mining. The solvent selection guide aids in the selection of greener solvents for reactions. These tools are designed to foster critical thinking. AI4Green4Students has additional pedagogical tools such as data management, collaboration, communication and project management (Figure S8). The details are given in the Supporting Information.

### AI4Green4Students in the Undergraduate Teaching Laboratory

#### 3rd Year Organic Chemistry Mini Lab Project

The third Year undergraduate laboratory teaching at the University of Nottingham comprises a mandatory project involving 4 weeks (4 × 1.5 days) of laboratory time as well as additional planning and report writing weeks. This course focuses on experiment design, laboratory practice, and developing team and communication skills. It covers chemical synthesis, catalysis, spectroscopy, and redox. Students review literature, plan and execute experiments, and write reports. A key project centers on the use of the Pd-catalyzed Suzuki-Miyaura coupling reaction^55^ to produce a library of biaryl compounds for use in screening and drug discovery. The AI4Green project, a variation of the original Suzuki-Miyaura project, was introduced to align with sustainable chemistry goals. This variation tasked students with modifying the reaction conditions of the given Suzuki-Miyaura reaction for the synthesis of a small series of biaryl compounds to improve green credentials such as energy efficiency, solvent selection, and waste generation. Data generated were analyzed to compare the effectiveness of different reaction conditions. This project had four objectives: documenting experiments, applying sustainability metrics, informing decision-making for improved sustainability, and learning how to form carbon-to-carbon single bonds. We adopt a framework for integrating green and sustainable chemistry, emphasizing four key principles: focusing on underlying chemical phenomena, gradually increasing complexity, using engineering practices for decision-making, and analytic tools to manage cognitive load through data organization and visualization.^11^

### Project Background

The Suzuki-Miyaura coupling reaction is pivotal in pharmaceutical drug manufacture, involving the cross-coupling of organoboranes with aryl halides using a transition metal catalyst, ligand, and aqueous base^56^ (Figure 2). It is the second most common reaction in medicinal chemistry,^57^ notable for its functional group tolerance and ability to form diverse carbon–carbon bonds. Despite mild conditions, it uses nonbenign solvents and precious metals, challenging sustainability. Thus, it can serve as a teaching tool for sustainable chemistry principles. Top of Form

**Figure 2:**
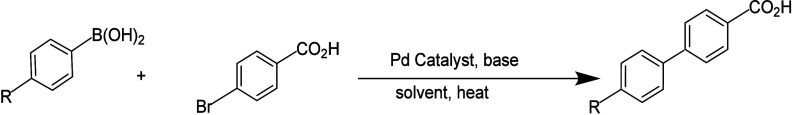
Reaction scheme for the Suzuki–Miyaura coupling.

The reaction methodology involves combining bromobenzoic acid and phenylboronic in a two-necked round-bottom flask, utilizing an aqueous sodium carbonate solution and the precatalyst stock solution at varying conditions. The Suzuki Method section in the Supporting Information includes a detailed reaction procedure.

### Implementation of AI4Green4Students in 3rd Year Chemistry Laboratory Class

Each student registered an account on the AI4Green4Students app and gained access to the ELN functionality upon joining a project group or being added by their instructor. This allowed tracking of each entry via usernames. The AI4Green project and the Year 3 project group, comprising six students and an instructor, were established. A dedicated project group page facilitated collaboration, while individual digital lab notebooks allowed students to enter experiment details privately, granting group members view-only access.

The web-based ELN could be accessed from any location and device, allowing students to add to their experiment record from the lab, home, or library. The lab provided 8 in. Android tablets with stylus pens for real-time data entry. Training materials, including videos, manuals, QuickStart guides, and other resources, were integrated into the app’s help page and regularly updated. The project marking scheme requires students to demonstrate teamwork, time management, safety, good laboratory practices, technical proficiency, knowledge, critical thinking, technical writing, and presentation skills, and the ELN has been designed to help them develop these skills.

Students completed four main tasks including learning sustainable chemistry, planning and designing experiments, documenting lab notes and generating reports. The general workflow and use of AI4Green4Students is shown in Figure 3.

**Figure 3:**
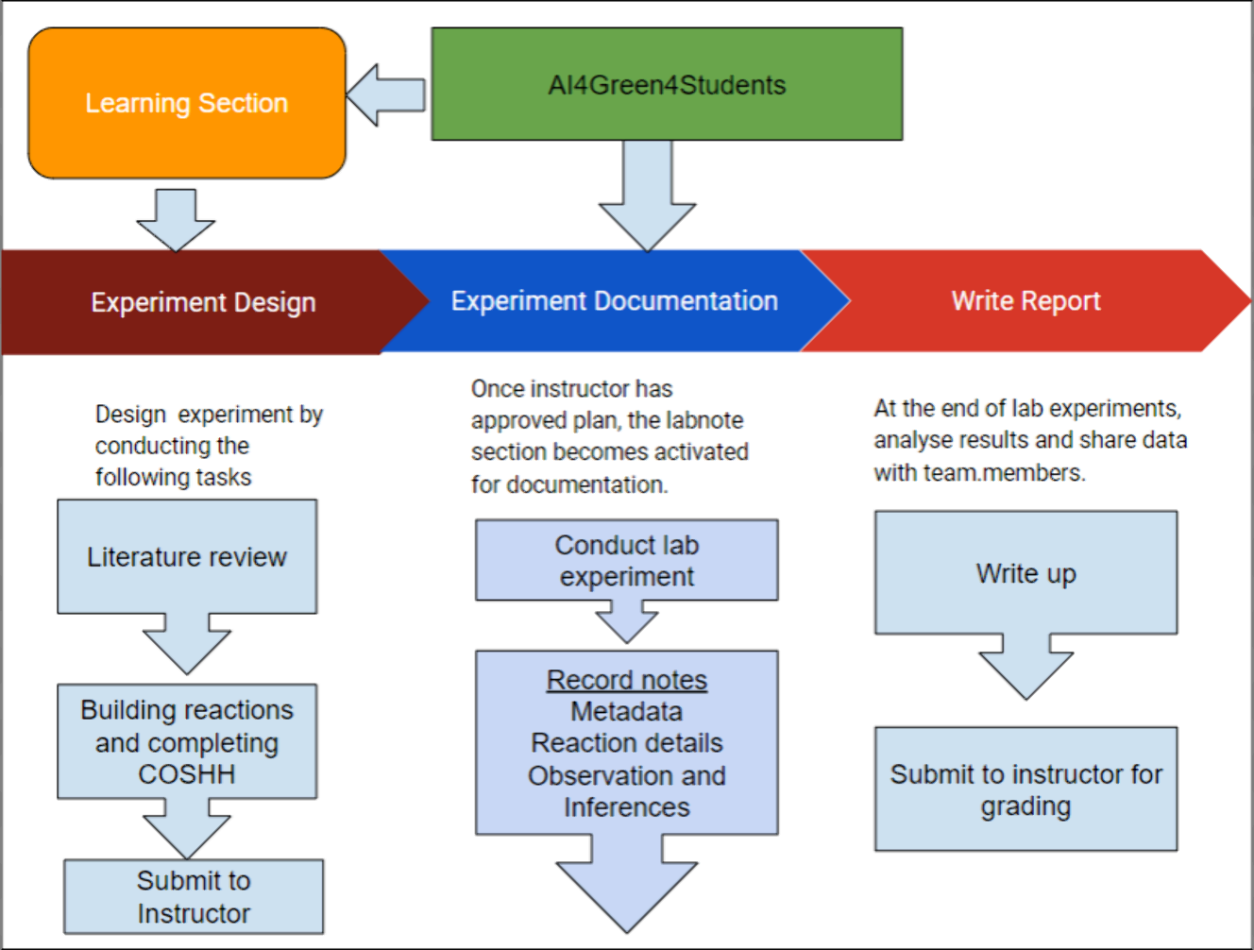
Workflow of AI4Green4Students showing the tasks completed by the students.

#### Learning Section

The Learning Section of the AI4Green4Students provides resources for learning sustainable chemistry. It also integrates an interactive quizzing tool to assess students’ green and sustainable chemistry knowledge (see Table 2). Complete questions with answers are shown in the Supporting Information.

**Table 2:** Sample Questions from the Sustainability Quiz

Question 1	Question 2	Question 3	Question 4
Which of the following are factors that affect the toxicity of a chemical?	Although a reaction may have an atom efficiency of 100%, it still possesses a large E-factor. What might be the reasons for this?	Which of the following are key principles in the functioning of a circular economy?	Which of the following is **not** a pillar of the triple bottom line?
			
(1) The route of exposure/ingestion.	(1) The selectivity may be low.	(1) Switch to using bioderived resources and eliminate the use of finite resources.	a. Society
(2) The concentration of the chemical.	(2) The E-factor is not derived from the atom efficiency.	(2) Keep products and materials in use.	b. Safety
(3) The duration of exposure.	(3) A large volume of organic solvent may be used.	(3) Prevent the degrading of natural systems by restoring nutrients to the biosphere.	c. Environment
			d. Economy
a. (1) and (2)	a. (1) and (2)	a. (1) and (2)	
b. (1) and (3)	b. (1) and (3)	b. (1) and (3)	
c. (2) and (3)	c. (2) and (3)	c. (2) and (3)	
d. All of the above	d. All of the above	d. All of the above	

The quiz, adapted with permission from the Sustainable Chemistry module, covers key topics such as chemical toxicity, green chemistry, circular economy principles, sustainability pillars, and green engineering. After completion, students receive an overall score and detailed feedback, highlighting correct and incorrect responses. This helps identify areas needing further review, allowing students to retake the quiz and improve their understanding of sustainable chemistry before progressing to practical applications.

#### Experiment Design and Planning

Students plan and design their experiments, highlighting the reaction conditions to be varied on a week-by-week basis before proceeding with the lab work. During this stage, students review journal articles to determine the appropriate procedures for their experiments. The literature review section within the AI4Green4Students planning section allows them to write a literature summary and attach relevant academic papers directly to their experiment plans. Next, students use the reaction-building tools (consisting of a Reaction Sketcher and Reaction Table) to create reaction schemes, visually representing the chemical reactions they plan to perform.

Students utilize the Reaction Sketcher (Figure 4) to draw reaction schemes or input SMILES strings. The system interfaces with the PubChem database to populate a Reaction Table (Figure 5) automatically with key details, such as the names of reactants and products, molecular weights, and densities. Students manually input additional relevant information, including hazard codes, hazard descriptions, physical forms, and quantities. The system provides feedback by displaying an ’incorrect answer’ warning when erroneous hazard details are entered, prompting students to reconsider their inputs. As the Reaction Sketcher does not support the inclusion of reaction conditions over the reaction arrow, students use the “Add Reagent” and “Add Solvent” functions to input the appropriate reagents and solvents. Solvents are color-coded according to Chem21 sustainability criteria, guiding students toward environmentally preferable options.

**Figure 4:**
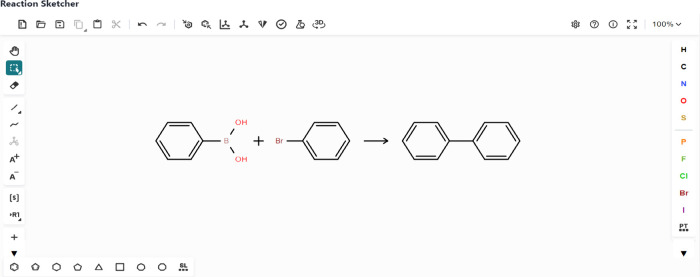
Integrated open-source Ketcher reaction sketcher for drawing reaction schemes.

**Figure 5:**
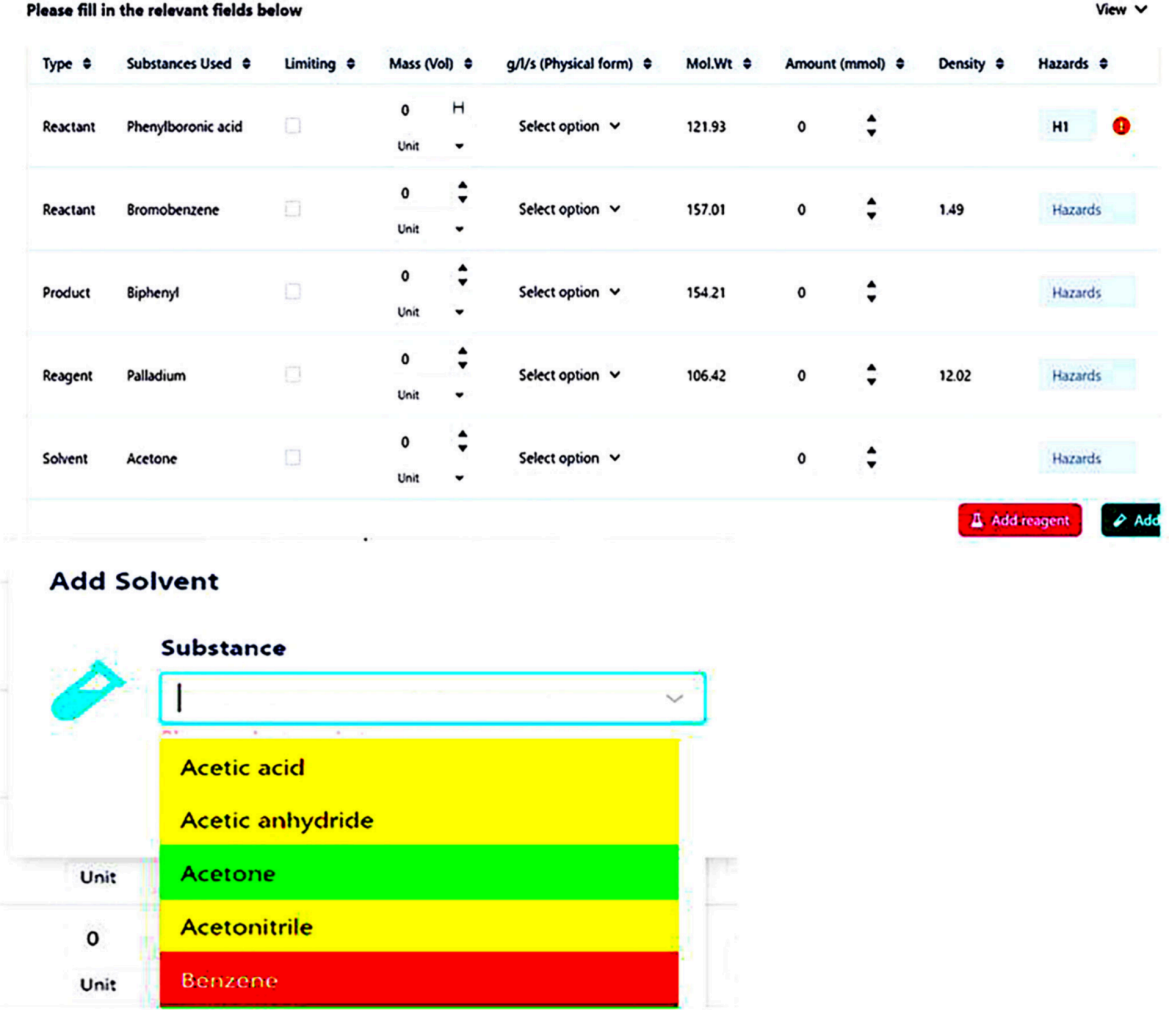
Reaction table showing solvent selection in a color-coded format.

Following the design phase, students complete the integrated COSHH forms to ensure compliance with health and safety regulations and finalize the experimental procedure, utilizing templates and guided forms. These forms help them systematically outline their objectives and methodologies,^27^ ensuring all critical aspects of the experiment are addressed. The submission button allows students to submit their plans to their instructor, who will assess and give feedback to them using the feedback features. Once instructors are satisfied with the students’ experimental plan, they can either approve it or request modifications. The lab notes section is only activated after the plan receives approval, ensuring that no experiments are conducted before addressing all health and safety concerns. Following plan approval, students utilize the lab notes section to document their experiments and the report section for detailed report writing (Figures S6 and S7).

#### Evaluation of Usability

Usability testing of the AI4Green4Students was conducted to assess the effectiveness of the ELN.^58,39^ Questionnaires and focus group meetings were employed to survey students to ensure the depth, credibility, and validity of the findings. While questionnaires provide broad, structured feedback from many respondents, focus groups offer deeper, qualitative insights through group discussions as well as contextual reasoning behind participant opinions.^59^ The questionnaires were designed and evaluated following the methodology of Venkatesh et al.^41^ Cronbach’s Alpha^60^ value of 0.7 (Table S1) demonstrates the internal consistency and reliability of the survey data. Open-ended questions regarding users’ experience were included to gather detailed feedback for the ELN formative evaluation.

A sample of survey questions from the Post-Study System Usability Questionnaire (PSSUQ)^61^ is shown in Table 3 and the complete questionnaire, detailing the question types together with the explanation of question labels and response options, is provided in the Questionnaire section of the Supporting Information.

**Table 3:** Sample Questions for Conducting a Usability Test

Section 2. Use and application of AI4Green4Students for Planning and Conducting Lab Experiments. Please answer the following questions in detail.
1. In how far did the app help in completing the COSHH form compared to the OneNote Notebook? If you have used anything other than OneNote Notebook please specify.
2. How did the app facilitate the planning of the experiment and writing a literature review before the actual experiment?
3. Did the learning contents, quizzes and hyperlinked Web sites help you in learning and applying sustainable chemistry?

The survey was conducted both before and after the development of AI4Green4Students. Questionnaires were administered using Microsoft Forms. Focus group meetings were audio-recorded via MS Teams and transcribed anonymously. Two categories of students participated: AI4Green Project students specifically recruited to test the app and non-AI4Green students to serve as the control group.

The non-AI4Green group was surveyed in the winter term, while the AI4Green group was surveyed in the spring term before the start, during the project execution, and after the project’s completion. Details on the survey’s framework and execution can be found in the Supporting Information. The survey results were evaluated after the survey period ended. Quantitative analysis was conducted and qualitative analysis was carried out with Nvivo.^62^

## Results and Discussion

### Eliciting User Requirements before the ELN Development

AI4Green project participants (*N* = 6) and nonparticipants (*N* = 6) took part in the initial survey, with nine out of 12 students responding. Of those, 80% expressed a desire for green and sustainable chemistry content to be integrated into the ELN and 90% requested collaborative features (Figure S10).

### Usability and Validity Test to Refine the AI4Green4Students Design

Upon implementation, participation in the focus group discussions and surveys was limited to students engaged with the AI4Green Project. At the time of evaluation, certain features of the ELN, such as lab notes and reporting functions were not fully operational, leading to incomplete survey responses. Questions relating to note recording and report writing were unaddressed. Students used the ELN for experiment planning, while continuing to rely on OneNote for lab note documentation.

Thematic analysis^63^ was employed to explore qualitative responses, revealing four key themes that highlight both the impact of the AI4Green4Students application and areas for improvement. Promoting sustainable chemistry was the most prominent, with users valuing the ELN’s ability to enhance awareness and understanding of sustainability in the chemical sciences. A word frequency search (Figure 6) supported this, identifying “metrics” as the most frequently used term, accompanied by related terms such as “sustainable”, “green”, “solvent”, and “calculating”. These findings suggest a strong emphasis on sustainability within the experimental processes recorded in the AI4Green4Students ELN.

**Figure 6:**
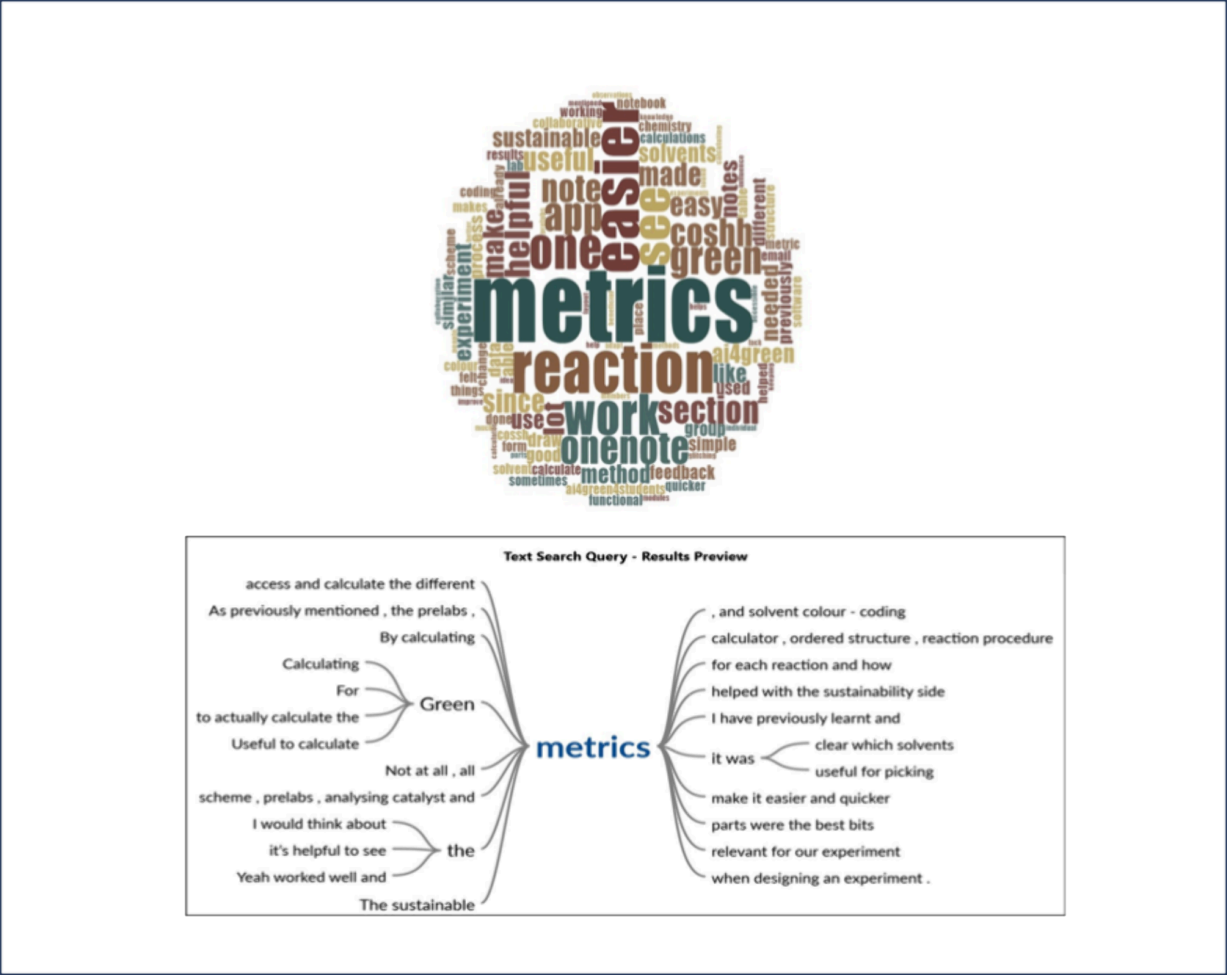
Word frequency chart and Search Tree Query showing that AI4Green4Students promoted learning and application of Sustainable Chemistry.

Student feedback on the ELN’s effectiveness in facilitating the learning and application of sustainable chemistry showed significant engagement with environmental concepts. Participants noted that the app provided clarity on the environmental impact of solvents, aiding their selection process. This was supported by the fact that all six project participants incorporated green metrics into their reports and presentations, using the calculations to inform their conclusions. In contrast, none of the six participants in the non-AI4green project group working under similar experimental conditions without the app referenced green metrics. This contrast highlights the ELN’s ability to embed sustainability within students’ workflow and promote environmentally conscious decision-making. A quantitative analysis of responses to closed-ended survey questions corroborated the app’s impact. As illustrated in Figure 7, all participants agreed that AI4Green4Students enhanced their understanding and application of sustainable chemistry principles.

**Figure 7:**
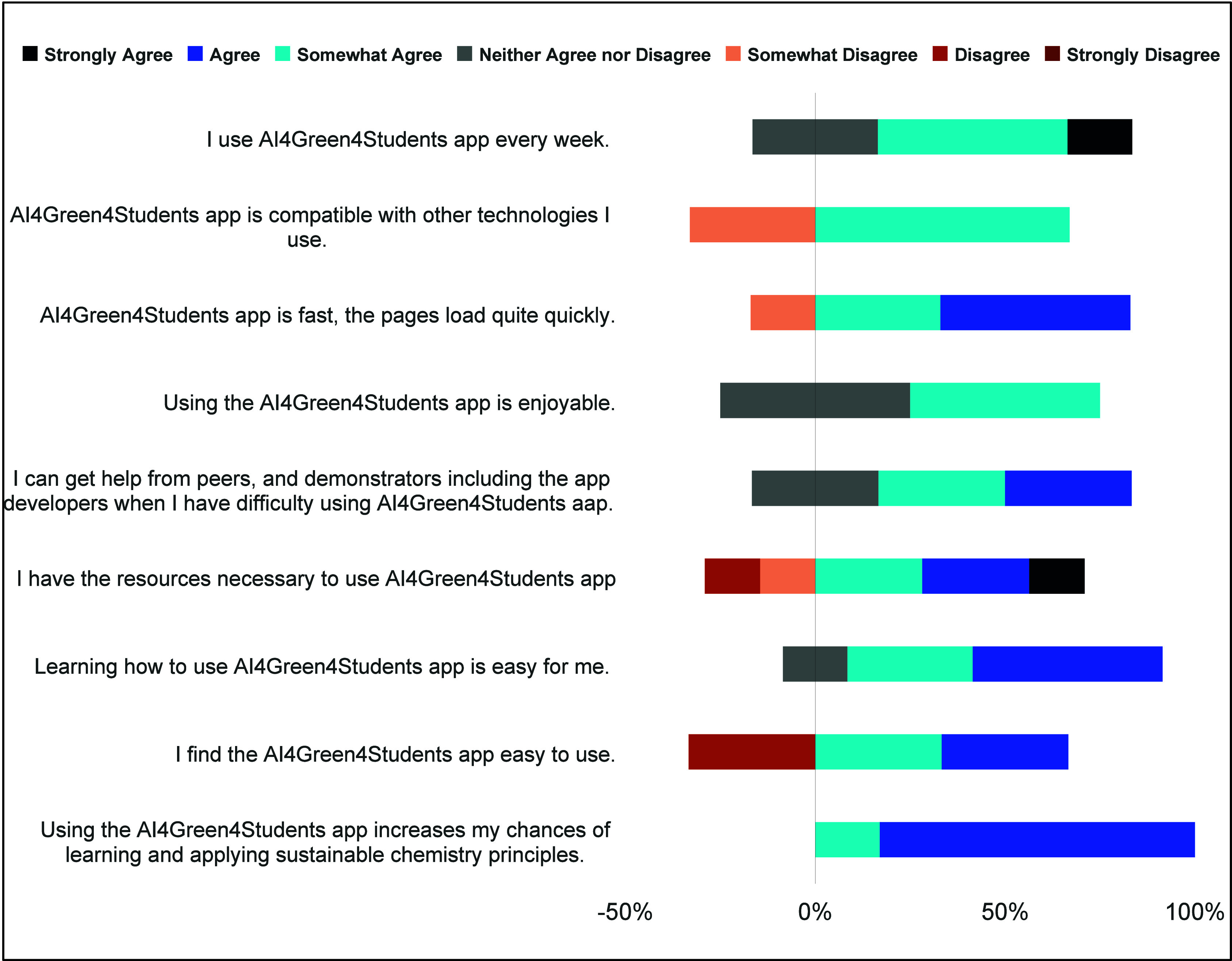
Responses demonstrating students’ positive attitude toward the adoption of AI4Green4Students.

The ELN’s implementation provided students with their first practical experience of evaluating chemical reactions based on sustainability metrics. This hands-on approach aligns with pedagogical principles that emphasize active learning for greater retention. Feedback indicated that the ELN facilitated more efficient learning by streamlining the error identification process for students and instructors. Instructors, in particular, found the ELN useful for quickly identifying planning errors.

A notable feature of the ELN was its simplified data entry and management system. Five of the six students reported that the ELN’s structured templates made complex chemical concepts more accessible. Comments such as “made a more structured view of what we needed to fill in and easy to draw reaction schemes” and “the ELN made it easier to complete the COSHH form as the tick boxes in the safety and waste disposal sections saved time” highlighted its time-saving benefits. However, some students found the mandatory fields in the structured templates burdensome. Despite this, four of the six students found the ELN easy to use, and three out of six felt supported by the training resources.

To evaluate AI4Green4Students further, students were asked to compare its features with OneNote, which is currently used in the teaching lab (Figure 8).

**Figure 8:**
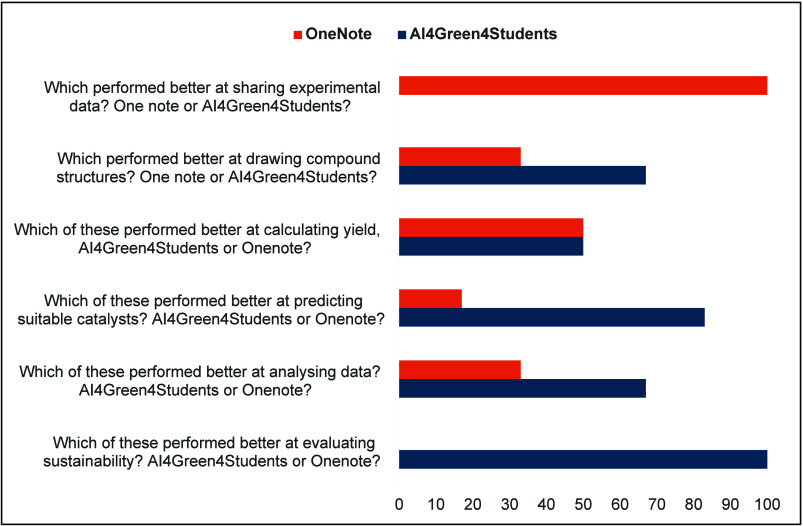
Comparison between AI4Green4Students and OneNote Notebook.

Four of the six students reported that AI4Green4Students outperformed OneNote in drawing compound structures, while five indicated it was better for catalyst selection, thanks to its sustainability element table. All six agreed that the ELN was superior in evaluating reaction sustainability. The app’s color-coded solvent selection feature, which ranks solvents based on health and environmental impacts, was appreciated. However, OneNote was considered better for sharing notes and recording observations, because at that time the Lab Note feature in AI4Green4Students was not operational.

Focus group discussions provided insights into why students ranked AI4Green4Students higher. One student remarked, “In the AI4Green4Students ELN, you have everything you need in one place. With OneNote, it is a bit disjointed”. Another student highlighted difficulties with OneNote’s flexibility for updating notes, saying, “You cannot go back and add information easily; I had to redo everything”. These reflections underscore the importance of a cohesive and intuitive digital tool in enhancing academic and laboratory workflows.

### Planned Improvements and Further Study

We gathered student feedback through the PSSUQ interviews and weekly input from their instructors. Positive feedback highlighted the structured templates, efficient feedback mechanisms, and tools for calculating green metrics. However, students expressed concerns during meetings. Issues with the Reaction Scheme feature included bugs that hindered drawing reaction schemes, leading many to use OneNote instead. The bugs were fixed and some students could use the feature successfully. Another concern was the excessive mandatory fields in the structured COSHH form, which students felt caused unnecessary data entry. Instructors, however, advised that these fields were necessary for comprehensive safety awareness. Hence, students were advised to enter “NA” when not applicable.

Collaboration was another key student request. While the development team had added a Group activity page, students wanted the ability to view each other’s experiment plans and notes. This feature has since been added for students within a given project group. One area of disappointment was the Lab Notes feature, which was not fully functional during lab experiments and only became available during report writing. Although students could not test it, instructors and developers have since confirmed that it is fully operational.

Survey results emphasized the need for further integration of sustainable practices in teaching laboratories. In response, the AI4Green4Students ELN is being refined based on student feedback, with most missing features now integrated. Plans are underway to expand the ELN’s use to other institutions and in the organic lab course, including adding new experiments and machine learning tools for data analysis, with future iterations monitored through surveys.

## Conclusion

The integration of digitalization and sustainable chemistry will educate and ingrain sustainable practices in students. However, challenges such as entrenched work culture, data security concerns, dense curricula, and limited resources have hindered broader adoption by academic institutions. This study illustrates how sustainable chemistry and enhanced data management have been incorporated into the chemistry teaching laboratory through the implementation of the AI4Green4Students ELN in an organic lab course for third-year undergraduates. Surveys conducted during and after the lab course gathered student feedback on their experiences with the ELN, informing future enhancements.

The survey results indicated that the ELN facilitated students’ evaluation of the sustainability of their lab practices using green metrics calculators and a solvent selection guide. It has also streamlined workflow management and improved data recording and management by offering structured templates, promoting efficient data entry, and enforcing safety standards through automated hazard verification. These improvements have led to a more organized and compliant experimental environment.

Furthermore, AI4Green4Students has positively impacted learning dynamics in the laboratory by enhancing collaboration and communication between students and instructors. The ELN’s functionality and integrated feedback mechanisms have enriched the learning experience by ensuring that experimental data are thoroughly documented and accessible. These capabilities support ongoing interaction and prompt feedback, which are essential for refining experimental methods and enriching the educational process.

The advancements made by AI4Green4Students in managing and conducting laboratory experiments highlight the transformative potential of digital tools in educational settings. The emphasis on sustainability aligns with global educational trends toward environmental responsibility. Future iterations will leverage user feedback to refine and expand the system. This includes making the ELN accessible to other institutions, integrating additional sustainability metrics such as life cycle analysis and toxicity tests, and adding new experiments and machine learning tools to enhance data analysis and report assessment.

The use of ELN described in this paper has proven effective for organic chemistry synthesis and can be readily adapted for other applications. It is fully operational and accessible via the following link: https://ai4g4s.app. A quick-start guide outlining the use of the ELN is provided in the Supporting Information. Additional instructional documents, including demonstration videos for various sections of the ELN, are available on the help page.

## Supplementary Material









## Notes

ELN design and evaluation, showing wireframes, survey instrument and analysis, sustainability cheat sheet (PDF, DOCX)
AI4Green4Students QuickStart Guide (PDF, DOCX)
