# Supplementary material for: AI4Green4Students: Promoting Sustainable Chemistry in Undergraduate Laboratories with an Electronic Lab Notebook
Source: J Chem Educ. 2025 Jun 5;102(7):2720–31. doi: 10.1021/acs.jchemed.4c01393 (PMC12243079; doi:10.1021/acs.jchemed.4c01393)
Supplement: Supplementary file 2 [file ed4c01393_si_002.docx]

Supporting Information- ELN design and evaluation

**AI4Green4Students: Promoting sustainable chemistry in undergraduate laboratories with an electronic lab notebook**

Peace C Nwafor ^a^, Shason Gurung ^b^, Philip van Krimpen ^b^, Lenka Schnaubert ^c^, Katherine Jolley ^a^, Samantha Pearman-Kanza ^d^, Cerys Willoughby ^d^, Jonathan D. Hirst ^a*^

^a^ School of Chemistry, University of Nottingham, University Park, Nottingham, NG7 2RD, United Kingdom

^b^ Digital Research Service, University of Nottingham, University Park Nottingham, NG7 2RD, United Kingdom

^c^ Learning Sciences Research Institute, School of Education, University of Nottingham, Dearing Building (C85), Jubilee Campus, Nottingham, NG8 1BB, United Kingdom

^d^ School of Chemistry and Chemical Engineering, University of Southampton, University Road Southampton SO17 1BJ, United Kingdom

* Email: jonathan.hirst@nottingham.ac.uk

*Corresponding author:

Tel: +44 115 951 3478

| FAX | : +44 115 951 3562 |
| --- | --- |

Email: [jonathan.hirst@nottingham.ac.uk (Jonathan D](mailto:jonathan.hirst@nottingham.ac.uk%20%20(Jonathan%20D). Hirst)

Contents

[**ELN Design and Development** 2](#_Toc197374926)

[Wireframe Designs 3](#_Toc197374927)

[Different Sections of AI4Green4Students 6](#_Toc197374928)

[Learning Section for Sustainable Chemistry 6](#_Toc197374929)

[Planning Section 11](#_Toc197374930)

[Report Section 14](#_Toc197374931)

[Lab note Section 17](#_Toc197374932)

[AI4Green4Students Pedagogical Features 19](#_Toc197374933)

[**ELN Implementation in the Undergraduate Teaching Laboratory** 22](#_Toc197374934)

[Suzuki Reaction Experimental Procedure 22](#_Toc197374935)

[Survey design 23](#_Toc197374936)

[Survey Instrument 24](#_Toc197374937)

[Survey Deployment 24](#_Toc197374938)

[Data Protection Declaration 24](#_Toc197374939)

[Questionnaire 25](#_Toc197374940)

[Pre-app deployment questionnaire for user requirements 26](#_Toc197374941)

[Post-app questionnaire to estimate the use and validity of AI4Green4Students 28](#_Toc197374942)

[**Survey Analysis** 32](#_Toc197374943)

[**Eliciting User Requirements Before the App Development** 32](#_Toc197374944)

[Student’s Report – referencing metrics 35](#_Toc197374945)

[Sustainability Cheat Sheet 36](#_Toc197374946)

**ELN Design and Development**

Following the initial information-gathering phase, a user interface (UI) wireframe was created using Figma to outline the navigation structure and ensure a smooth user experience. This wireframe included connectivity to both internal and external databases and the navigation pathways. The Electronic Laboratory Notebook (ELN) was developed with a React framework for the front end and C# for the back end. A PostgreSQL relational database was chosen for its ability to handle complex queries and relationships, particularly for storing user information and chemical reaction data. The AI4Green4Students is sectioned into four: Learning Section, Planning Section, Lab note Section and Report Section. Some of the key ELN features include a reaction builder and sustainability calculator.


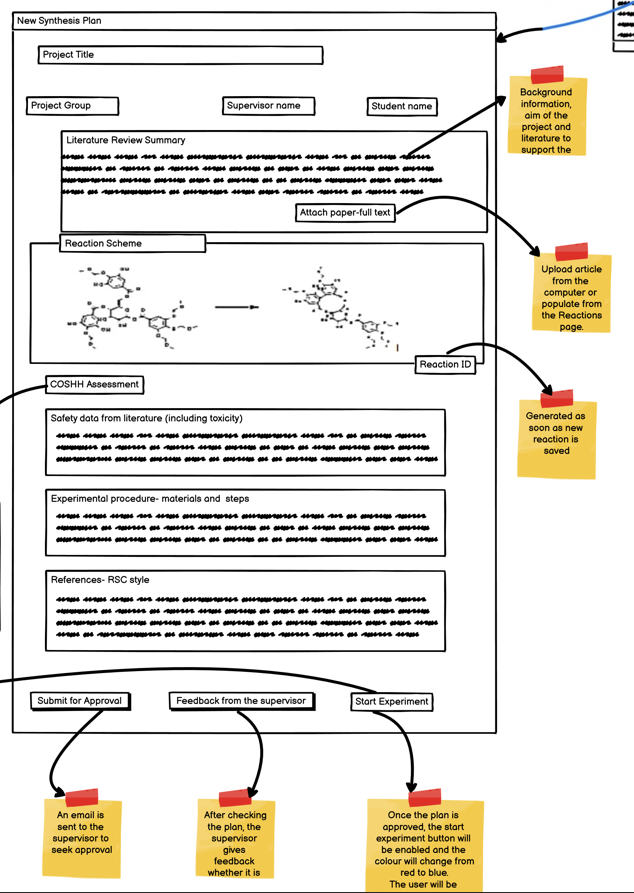
Wireframe Designs

**Figure S1** Wireframe Design of the Experiment Planning Section

**Figure S2** Wireframe Design of the Lab Note Section


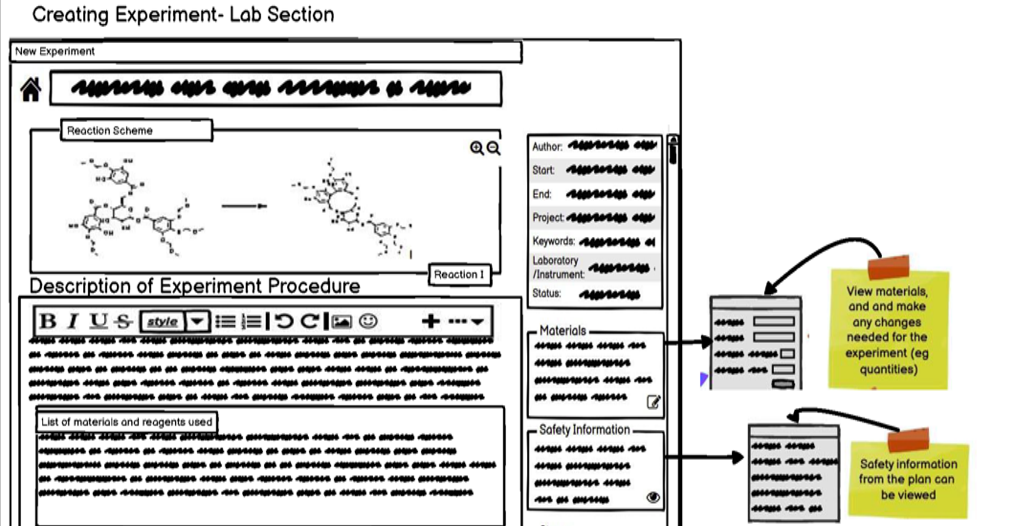

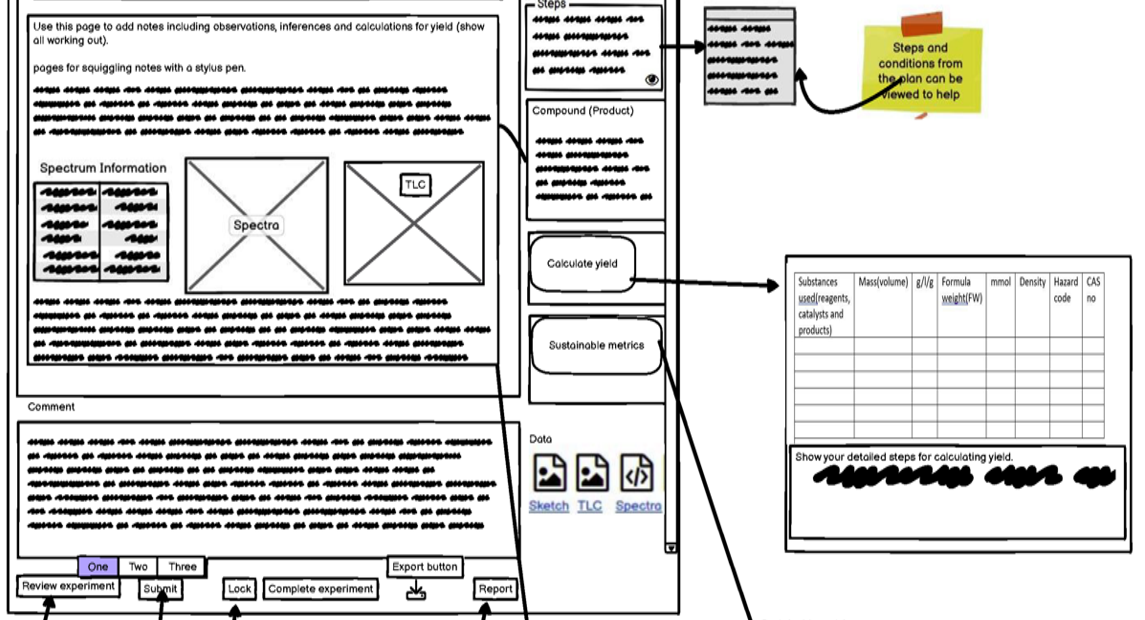


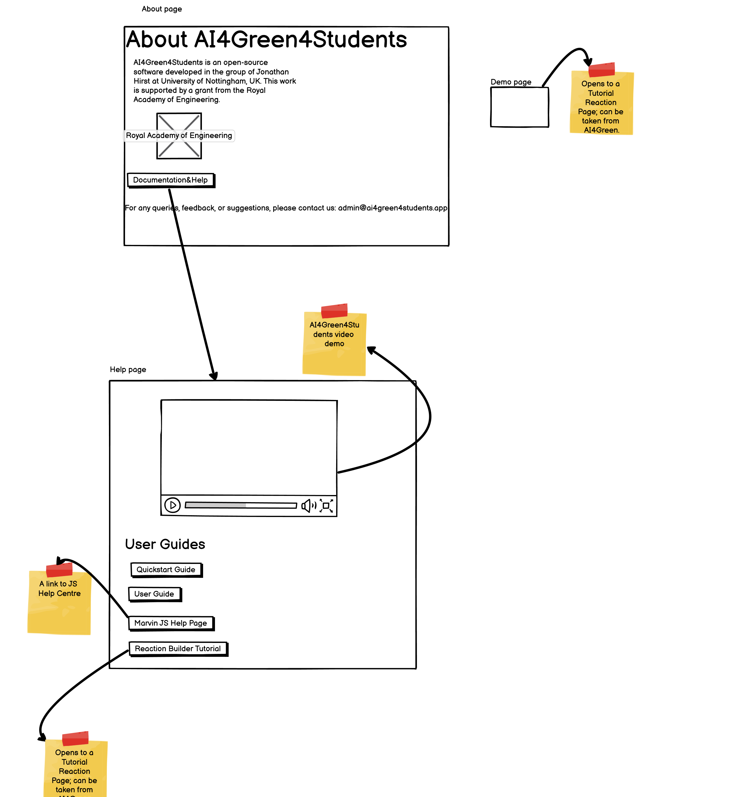


**Figure S3** Wireframe Design of the About Page of the

Different Sections of AI4Green4Students

Learning Section for Sustainable Chemistry

Sustainable chemistry learning resources are provided in the Learning Section of AI4Green4Students. They are useful for bridging knowledge gaps in sustainable chemistry, particularly for students who have not completed the Sustainable Chemistry Module in earlier years. It provides information on green chemistry, along with links to external sources like the American Chemical Society (ACS) for further reading.

**
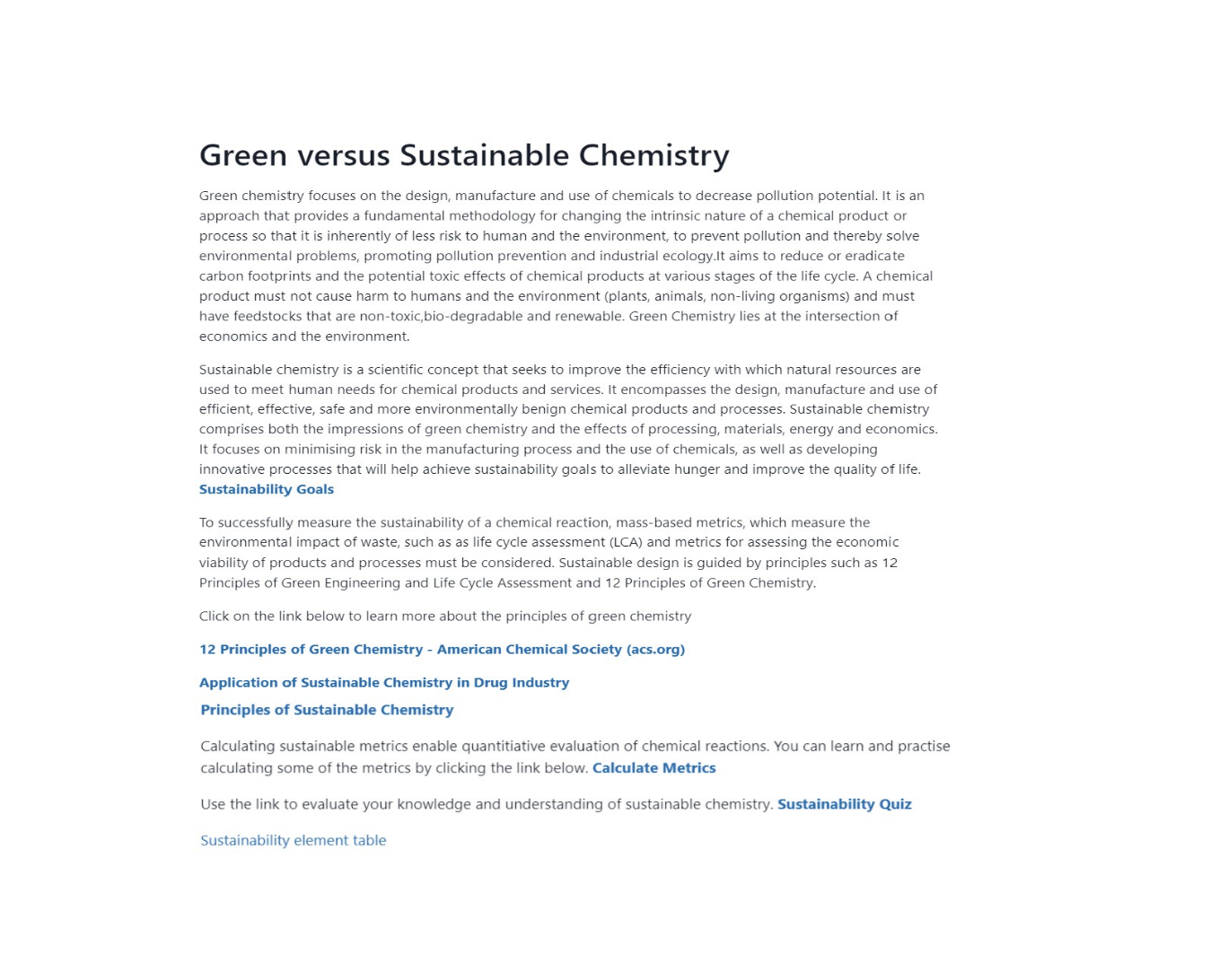
**

**Figure S4** Overview of Sustainable Chemistry content and external resource

An interactive quiz, adapted from the Sustainable Chemistry Module, covers topics such as chemical toxicity, green chemistry principles, circular economy, sustainability pillars, and green engineering. The quiz features multiple-choice questions, with points awarded based on question difficulty. Each question offers multiple choices, with 2 points awarded for higher-order reasoning and 1 point for single-answer questions. After completion, students receive a score and detailed feedback, identifying areas for improvement. This enables them to retake the quiz and strengthen their understanding before applying the concepts in practical settings. A score of 80% shows a good knowledge of sustainable chemistry. Below are the multiple-choice quiz questions with the correct answers marked with a tick.

**Sustainability Quiz**

1. **Which of the following best describes green chemistry?**
2. Consideration of the environmental, societal and economic spheres when discussing human development.
3. Design of chemical products and processes that reduce or eliminate the use and generation of hazardous substances.🗸
4. Businesses and governments should include social and environmental concerns in assessing their performance along with their usual economic concern.
5. Development and commercialisation of industrial products and processes that are economically feasible, minimise material and energy expenditure, and reduce the risk to human health and the environment.
6. What is the atom economy of the reaction shown below?


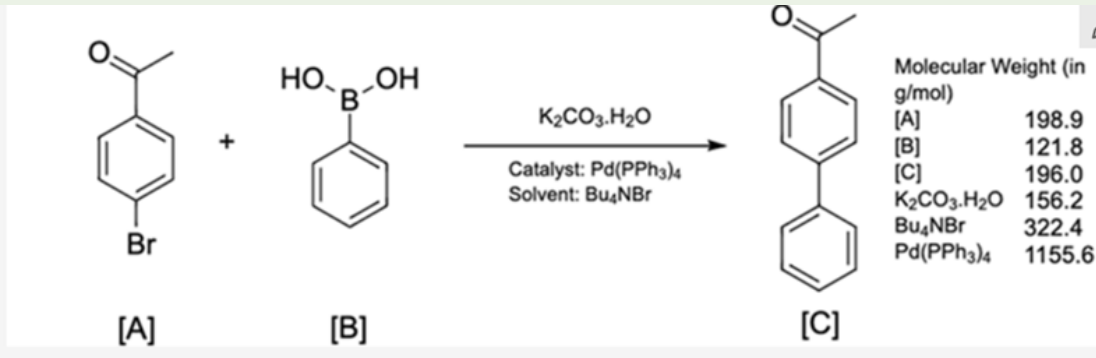


a. 10.0%

b. 20.2%

c. 41.1%🗸

d. 62.1%

3. What does a low value of LD50 signify?

a. An energy-inefficient process.

b. A long time for a chemical to degrade within the environment.

c. A short lifetime of a catalyst.

d. A highly toxic chemical.🗸

4. Within the 12 principles of green chemistry, principles 1, 2 & 8 shown below can be thought of primarily contributing to which goal?

1. Prevention

2. Atom economy

8. Reduce derivatives

1. Incorporating atoms into products more efficiently.🗸
2. Maximising energy efficiency for a given process.
3. Monitoring a given process for waste.
4. Use of more environmentally benign materials.

5. Which of the following most accurately describes the Sustainable Development Goals (SDGs)?

1. Various modes of measurement that allow for the determination of the efficiency of a chemical process.
2. A methodology of assessing and tackling problems with large, complex system that sees the interconnectedness of the parts to form a whole (system).
3. A set of principles proposed by the UN, to be followed by every member state, in order to achieve sustainability by 2030.🗸
4. They are a universal call to action, that ensures access to good health, education and clean energy for everyone.

6. Which of these is **not** a key principle of Green Toxicology?

1. Encourage stakeholders to support the idea of green chemistry.
2. Zero animal testing.🗸
3. Developing methods to do experiments virtually
4. Making testing sustainable.

7. Based purely on the information below, which of the following processes is the “greenest”?

1. A 5-step reaction with 100% conversion and 80% selectivity per step.
2. A single-step reaction with 100% conversion but 60% selectivity.
3. A 7-step reaction with 65% conversion and 95% selectivity per step.
4. A single-step reaction with a 50% conversion but 80% selectivity.🗸

8. Which of the following are factors that affect the toxicity of a chemical?

(1) The route of exposure/ingestion.

(2) The concentration of the chemical.

(3) The duration of exposure.

1. (1) and (2)
2. (1) and (3)
3. (2) and (3)
4. All of the above🗸

9. Which of the following best describes green chemical engineering?

1. Commercialization of industrial processes that minimize material and energy expenditure.
2. Commercialization of industrial processes that are economically viable.
3. Commercialization of industrial processes that reduce or eliminate the risk to human health and the environment.
4. All of the above.🗸

10. Although a reaction may have an atom efficiency of 100%, it still possesses a large E-factor. What might be the reasons for this?

(1) The selectivity may be low.

(2) The E-factor is not derived from the atom efficiency.

(3) A large volume of organic solvent may be used.

- 1. (1) and (2)
  2. (1) and (3)
  3. (2) and (3)
  4. All of the above🗸

11. Which of the following are among the 12 Principles of Green Chemistry?

1. Design commercially viable products.
2. Chlorinated solvents need to be banned from chemical development.
3. Use stoichiometric additives to improve reactions’ selectivity.
4. Use warning indicators via real-time monitoring of reactions.🗸

12. What are the limitations of the E-factor as a green chemistry metric?

(1) it traditionally does not account for energy inputs or process water

(2) it does not account for solvents and auxiliaries

(3) it does not say anything about how hazardous the waste is

1. (1) and (2)
2. (1) and (3)🗸
3. (2) and (3)
4. All of the above

13. Which of the following facts could be arguments in favor of nuclear power as a more sustainable method of power generation than solar farms?

(1) Solar farms require more land than nuclear power stations to produce a given amount of energy.

(2) Operation of a nuclear power plant requires a skilled and varied workforce for its lifetime.

(3) Nuclear power stations require fewer materials to produce than solar farms.

1. (1) and (2)
2. (1) and (3)
3. (2) and (3)
4. All of the above🗸

14. A major soft drinks manufacturer switches their product bottles from green to clear plastic on the grounds that it will be easier to turn the materials back into new bottles. Which of the following principles of green chemical engineering were the company addressing by making this change?

(1) Meet Need, Minimize Excess.

(2) Design for Separation.

(3) Minimise Materials Diversity.

1. None of the above
2. (1) and (2)
3. (1) and (3)🗸
4. (2) and (3)

15. The three statements below apply to the development of a partly metabolized new drug. Which of the following statements is correct?

(1) is financially viable

(2) has a safe design strategy and manufacturing process

(3) in silico tools were used to predict its toxicity

1. The development is sustainable because it aligns with the application of predictive toxicology.
2. The development is sustainable because a sustainable development of a drug does not primarily focus on its financial viability.
3. The development may not be sustainable because not all aspects of the triple bottom line have been considered.🗸
4. The development is sustainable because it’s beneficial for the consumers and the environment in addition to being economically viable for manufacture.

16. Which of the following is **not** a pillar of the triple bottom line?

1. Society
2. Safety🗸
3. Environment
4. Economy

17. Which of the following is **not** an example of a circular economy?

1. Sharing of products that are used less frequently, such as drills or cars.
2. A company using recycled plastic to produce disposable drinking cups.🗸
3. Recycling plastic into pellets for the manufacture of new plastic products.
4. Refurbishing an unusable car by building and installing a new engine, so that the car may be driven again.

Planning Section

This section helps students complete all the pre-lab tasks, including designing and planning experiments, completing the COSHH form and submitting it to the instructor. **Figure S5** **A&B** below shows different parts of the planning section.

**
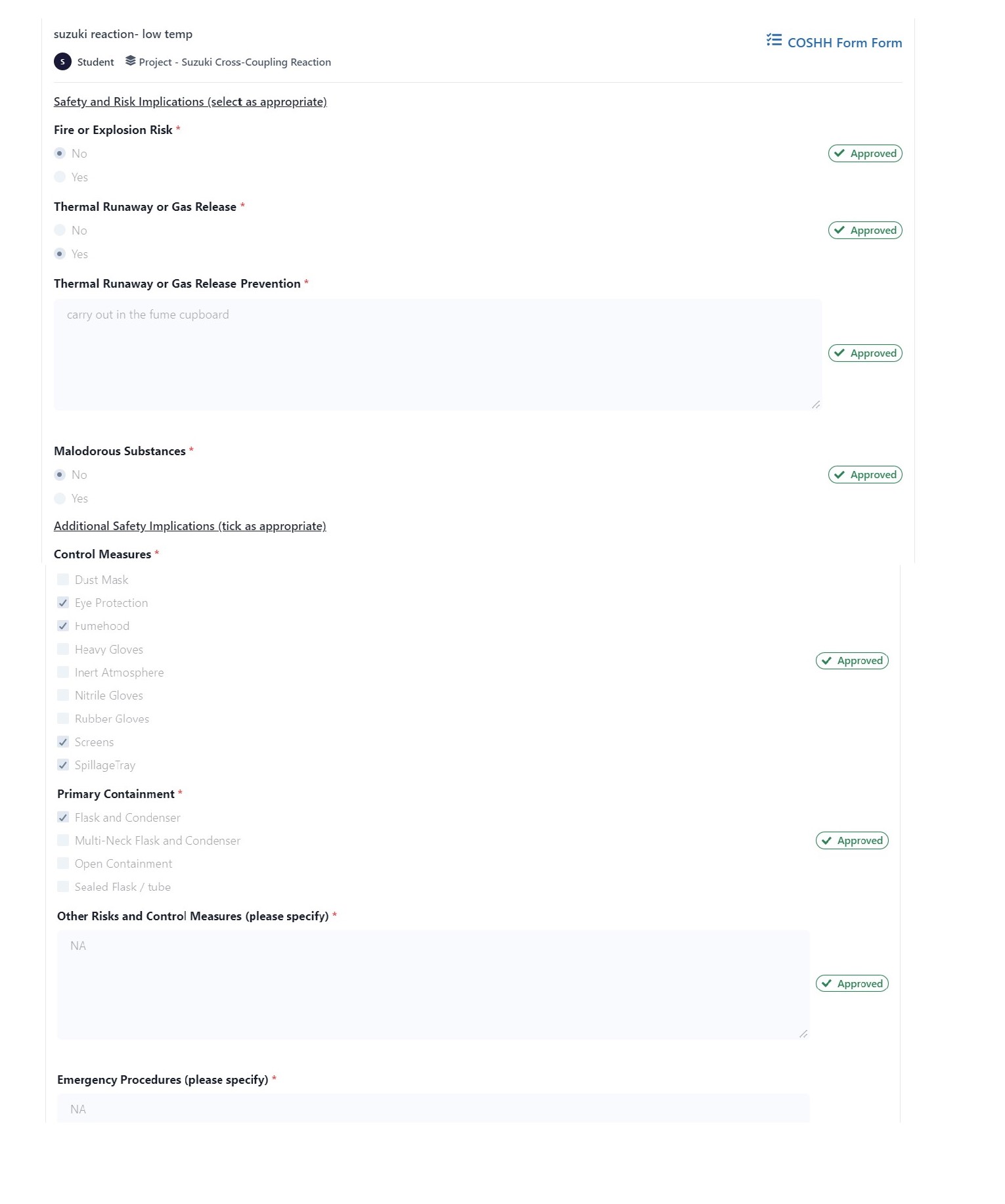
Figure S5** (**A)** Planning Section- COSHH Form for recording health and safety details of reaction.

**
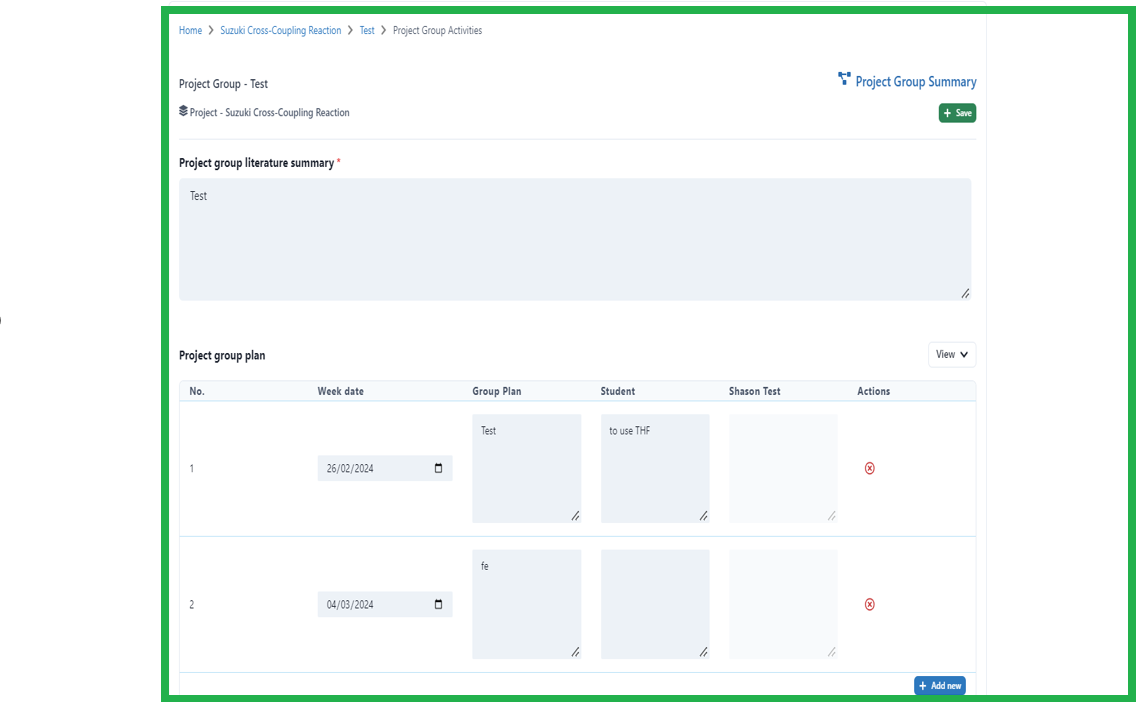
**

**Figure S5 (B)** Planning Section- Project management template for peer collaboration.

Report Section

Students utilize this section to write a report of the experiment. The report section (**Figure S6** A-C) becomes accessible as students conduct their experiments, allowing them to begin compiling their findings concurrently. The lab notes remain editable until the conclusion of the lab period, after which they are locked. This flexibility enables students to refine their experimental records as needed before finalizing them. The report section provides structured templates for organizing experimental results, data analysis, and conclusions, ensuring all key elements are addressed. Students can also attach supplementary materials, such as graphs, images, and references. Once completed, the report can be exported in Word format and submitted for grading via the institution’s virtual learning environment, Moodle.

.
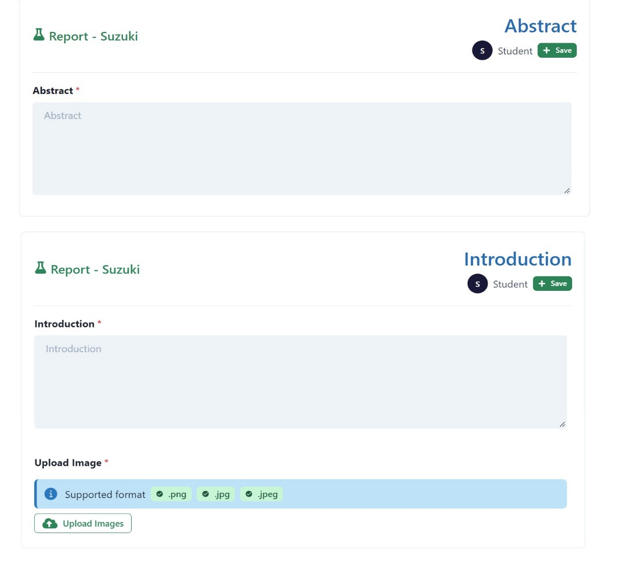


**Figure S6 (A)** Report Section – Introduction part.

**
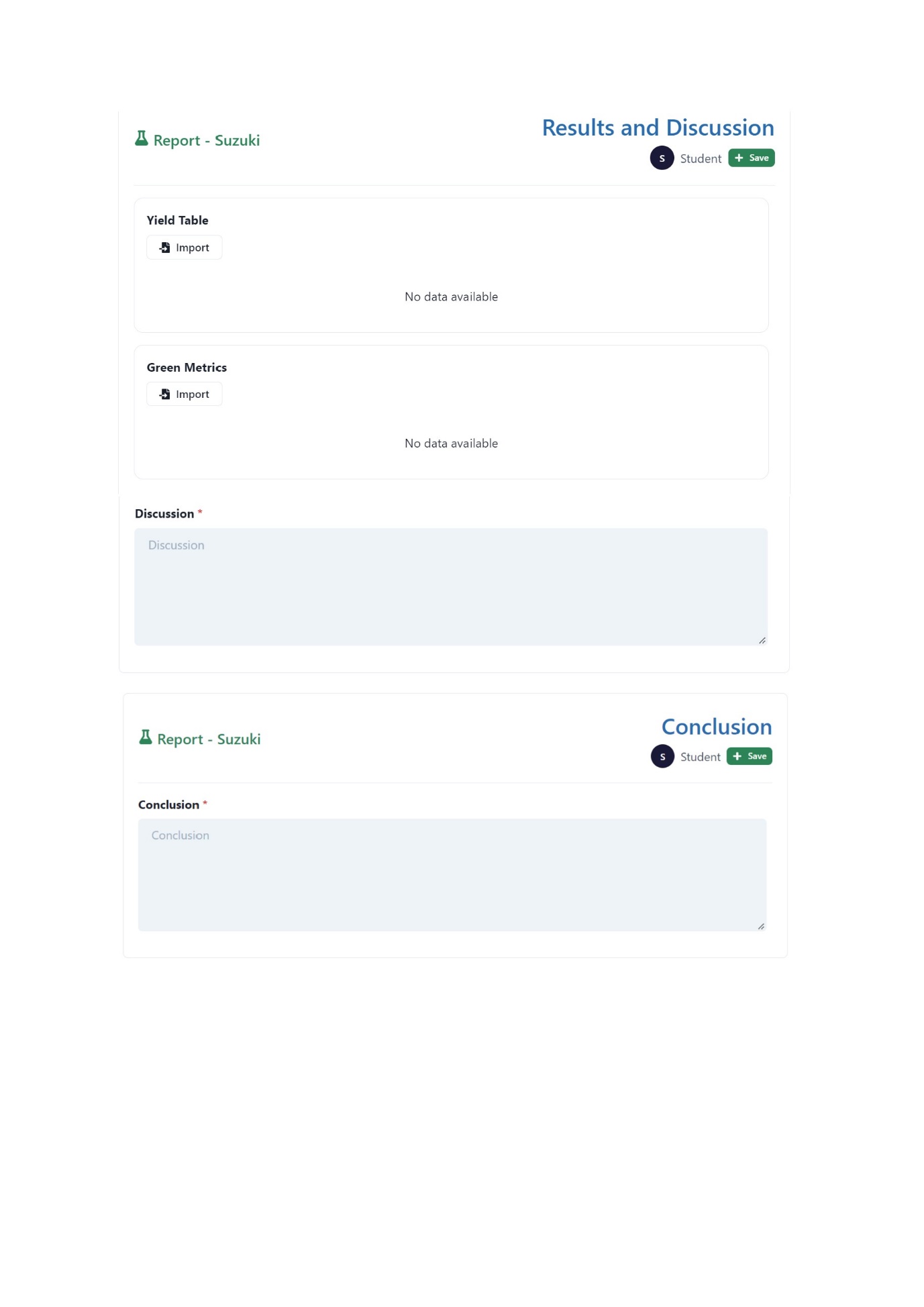
Figure S6 (B)** Report Section – Result and discussion part.


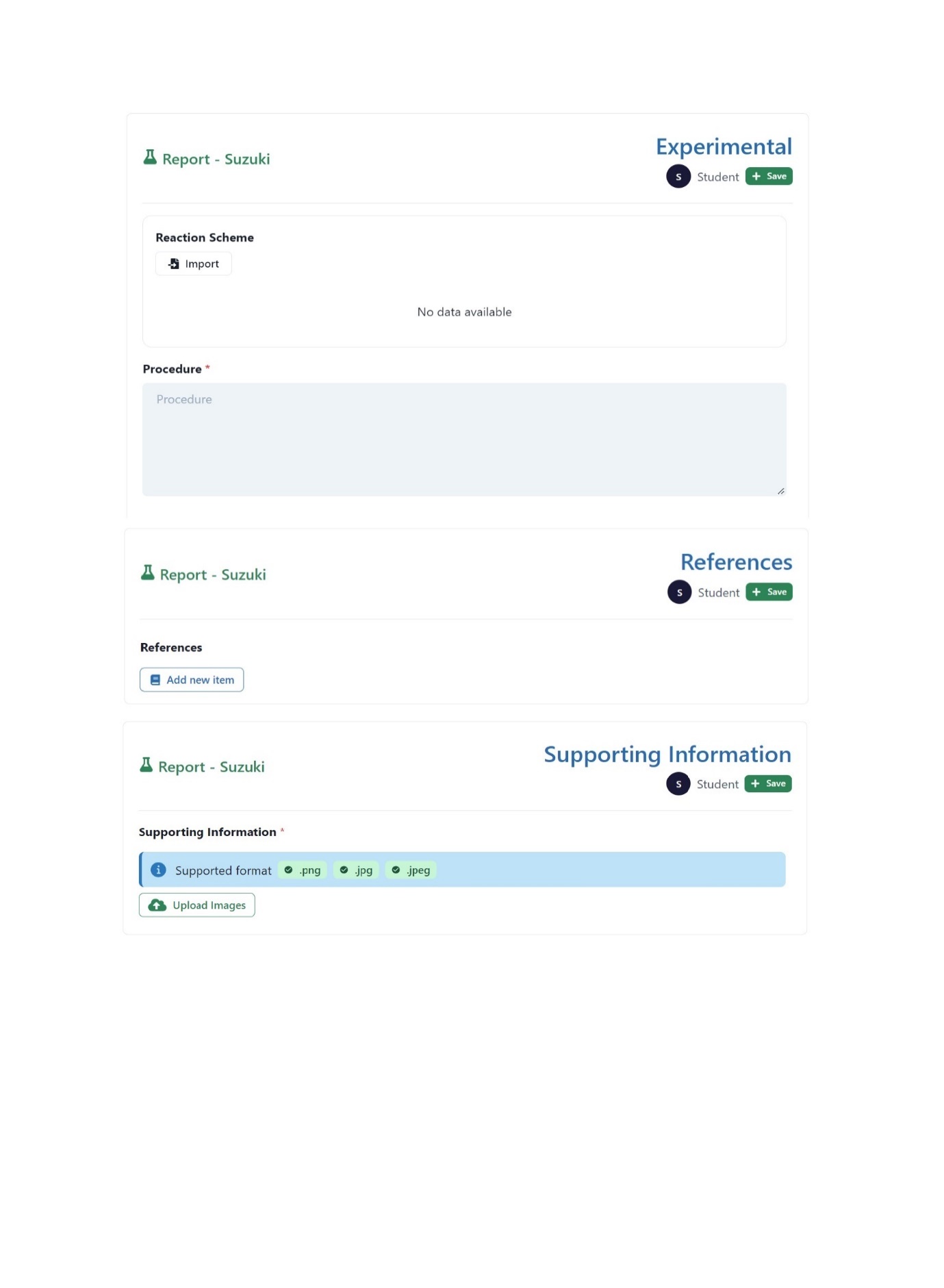


**Figure S6 (C)** Report Section – Reference part.

Lab note Section


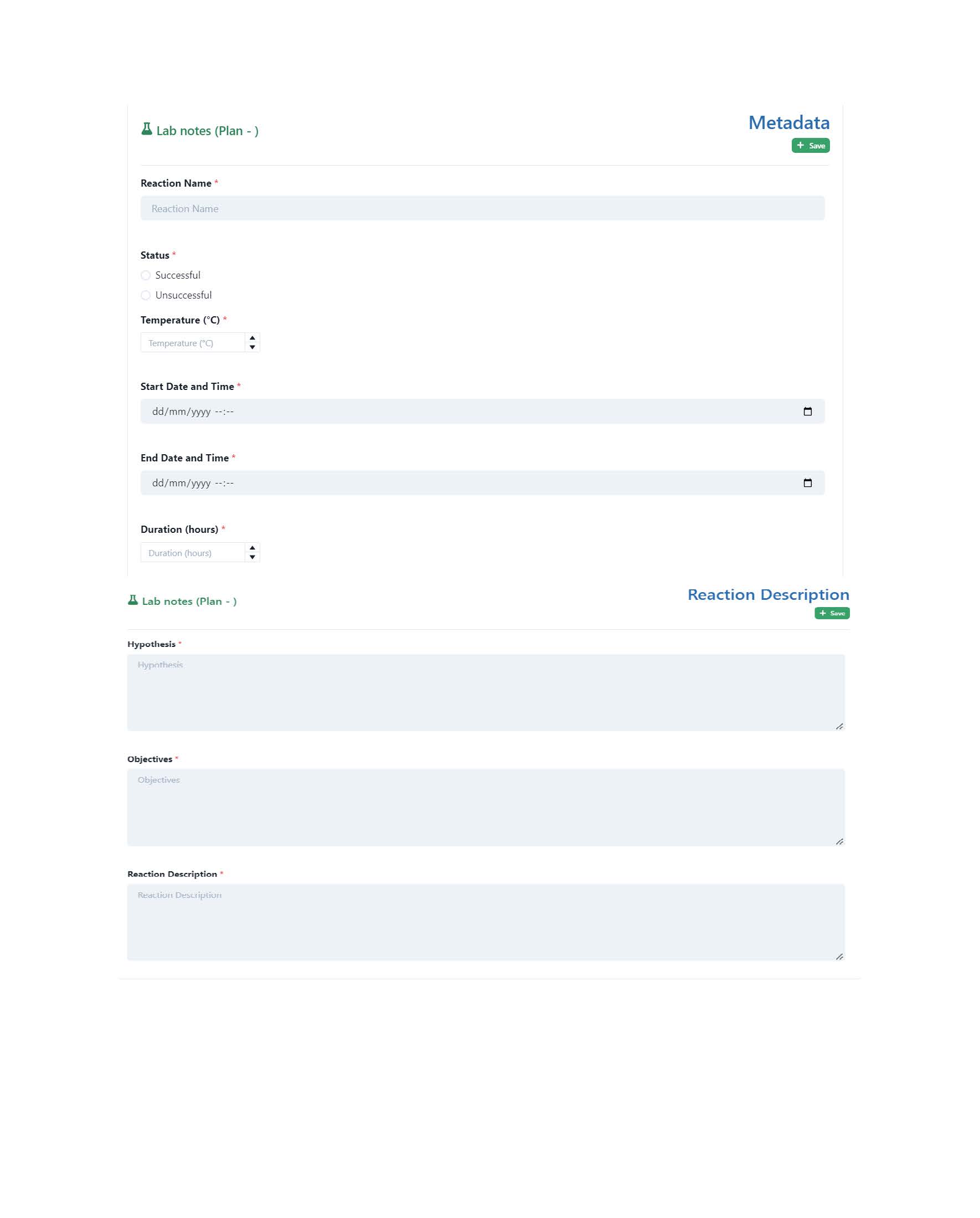
This section is a student's lab note but in a digital format. It is used to document experiments in real time (See **Figure S7 A&B**).

**Figure S7 (A)** Lab note Section showing templates for recording reaction details.

**
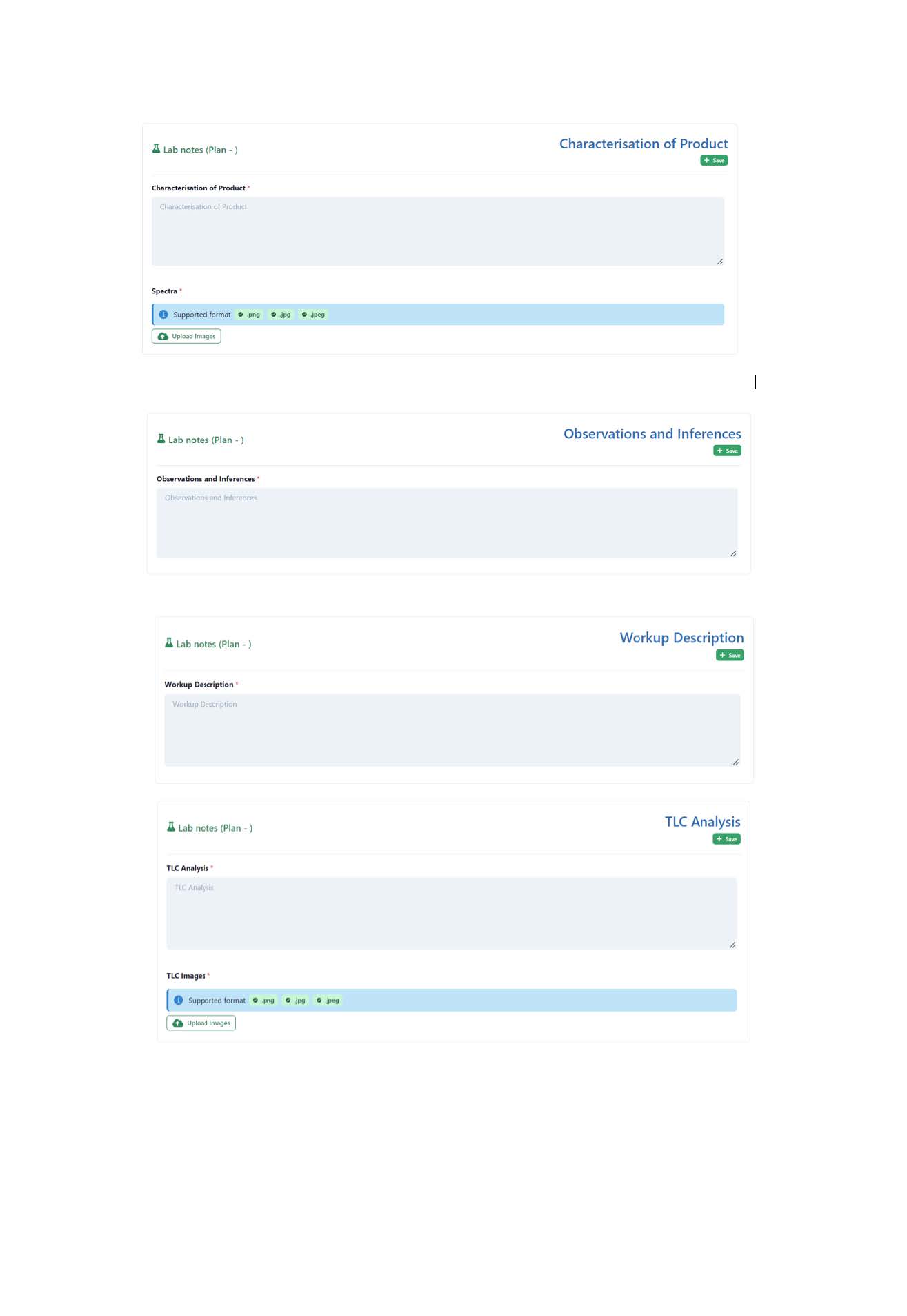
Figure S7 (B)** Lab note section showing templates for product characterization and purity test.

AI4Green4Students Pedagogical Features

**Data Recording and Management**

Proper documentation of data is essential for ensuring data is FAIR^1^. AI4Green4Students offers a centralized location for students to input, store, and organize their data, ensuring easy accessibility and management. Utilizing cloud-based technology, the application guarantees secure data storage, accessible from any device, thus mitigating risks of physical data loss and facilitating continuous work across various locations. The app’s search functionalities enable the retrieval of experimental data by structures or reaction names, enhancing data findability.

The app’s interface streamlines data entry, featuring auto-save and drop-down menus to minimize errors. The OneNote Notebook currently used for documentation in the teaching laboratory lacks structured data entry capabilities. AI4Green4Students offers templates for various data types, including experiment planning and report writing. Each app section (see **Supplementary Information)** contains detailed subsections: the planning section includes literature reviews, reaction schemes, safety data, and experimental protocols, while the lab notes section provides fields for metadata, reaction summaries, yields, green metrics, TLC analysis, purification steps, observations, and inferences. This structure supports accurate recreation of reactions. The report section covers abstracts, introductions, experimental details, results and discussion. AI4Green4Students improves data handling accuracy and efficiency, providing students with a reliable method for managing their academic and research data.

**Communication and Collaboration**

ELNs can enhance student collaboration and communication^2^. AI4Green4Students integrates features for interaction, data sharing, experiment planning, and collaborative project work. The shared notebook functionality allows group members to document individual observations and results on a unified platform, ensuring data capture and accessibility for all participants. This should promote teamwork and enrich the learning experience through peer-to-peer interaction and feedback, allowing students to review each other's contributions, offer constructive criticism, and gain insights from different perspectives.

AI4Green4Students also incorporates communication tools for information exchange among students, instructors, and the system. Integrated feedback mechanisms enable prompt instructor feedback and interventions. The 'Request Change' feature allows instructors to request modifications directly within the application, while embedded comment functionalities provide detailed, context-specific feedback. Automated notifications ensure students and instructors are informed about updates and changes.

The system's feedback capabilities also provide real-time educational support during experiment planning and execution. For example, incorrect hazard codes entered on the COSHH form are flagged with a warning, prompting students to review and correct entries, thereby supporting critical thinking and accurate documentation. These features collectively enhance project management, collaboration, and the overall educational experience by promoting accurate and reflective learning processes.

**Project Management**

The organizational structure plays a crucial role in project management by shaping the processes of communication, coordination, and decision-making^3^. AI4Green4Students employs a project group structure to facilitate project management through defined roles, responsibilities, and workflows. Each project group, termed a Projectgroup, contains two workbooks: Project Group Activities and the Projectbook. The Project Group Activities workbook enables collaborative project planning and coordination. At the same time, the Projectbook serves as a digital lab notebook for pre-lab tasks, real-time experiment documentation, and report compilation.

Three roles within a Projectgroup include instructors (Projectgroup owners with full permissions), demonstrators (restricted access for review and assessment), and students (limited permissions). This structure enhances accountability and efficient resource allocation (**Figure S8**). Reactions within the Projectgroup are shared exclusively among its members, ensuring data privacy. Additionally, this structure provides a system for peer support and managing deadlines, enabling detailed planning, task assignment, and progress monitoring


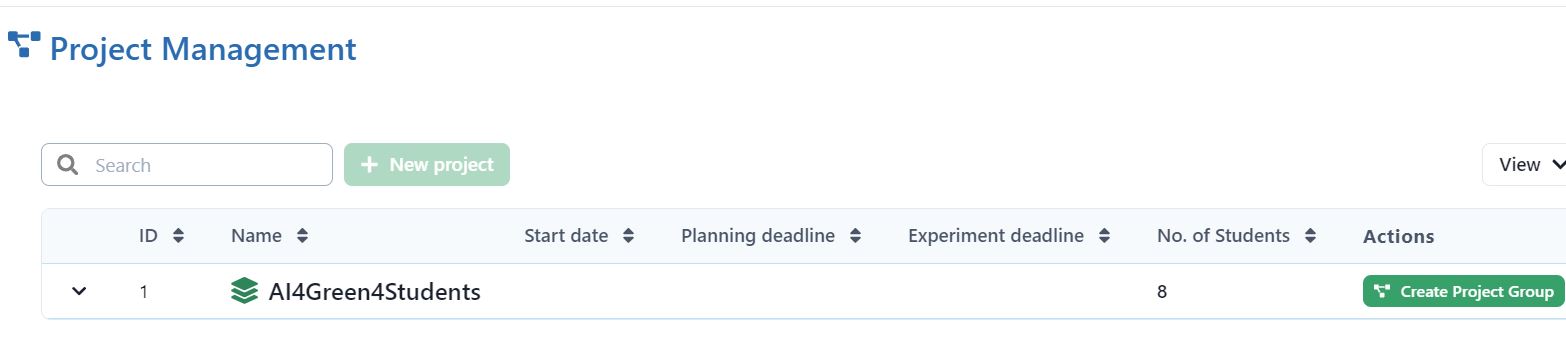

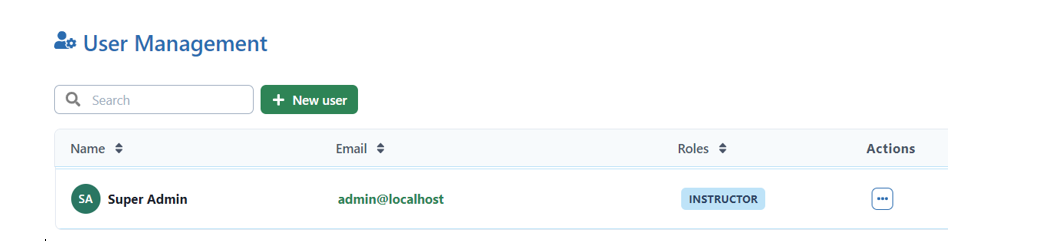


**Figure S8** User management features showing different roles; Project management feature for creating projectgroups.

**ELN Implementation in the Undergraduate Teaching Laboratory**

The ELN was integrated into the Year 3 MSci Chemistry Laboratory class, where students conducted Suzuki-Miyaura reactions under varying experimental conditions. Sustainability criteria were assessed using integrated sustainability metrics within the ELN. Over four weeks, students investigated variations in the Suzuki reaction parameters to optimize the synthesis of biaryl compounds under "greener" conditions. Key variables such as temperature, solvent type and volume, catalyst loading, and other relevant factors were altered to evaluate their impact on the reaction performance. Students utilized the AI4Green4Students ELN as a digital platform for recording reaction conditions and experimental outcomes, including yield, reaction time, and the calculation of pertinent green chemistry metrics. At the end of the laboratory experiments, they compiled and analyzed these data, comparing the effectiveness of various experimental conditions.

Students were given 10-inch Android tablets with which to access ELN in the lab, design experiments and document notes. Feedback was collected from students to evaluate the extent to which the ELN facilitated the application of sustainable chemistry principles and its overall impact on teaching and learning outcomes.

Suzuki Reaction Experimental Procedure

**Suzuki Cross-Coupling Reaction**

**Reaction Scheme**

To a 100 mL 2-necked round bottom flask containing a stirrer bar and thermometer was added 4-bromobenzoic acid (1.50 g, 7.5 mmol) and 4-methyl-phenylboronic acid (9 mmol, 1.2 equiv). To this was added aqueous sodium carbonate solution (1M, 3 equiv). Heat the stirred solution to between 70 and 80 °C (internal temperature). To the heated, stirred solution, was added the pre-catalyst solution (0.25mM, 0.1 mol%)^4^. The reaction progress was monitored by TLC every 30 minutes. A small aliquot of the reaction solution (0.5-1 mL) was acidified to pH1-2 by addition of 1M HCl aq. To this was added EtOAc (0.5-1 mL) and the solution mixed and allowed to settle to two phases. The EtOAc phase was analysed by TLC (5% MeOH in DCM).

Once the reaction is complete, the solution was cooled to <10 °C (ice/water bath) and acidify to pH 1-2 by careful addition of aqueous HCl (1 or 2M) to minimize effervescence. The precipitated product was collected by vacuum filtration (buchner funnel or glass frit) and wash with a small amount of ice cold *iso*-propanol. The crude solid was acidified by addition of 10 mL of 1M HCl aq and the mixture stirred and heated to 70 ^o^C. Isopropanol was slowly added to the solution, maintaining a temperature of 70 ^o^C until all material dissolves, before cooling to initiate crystallization. The recrystallized product was collected as a white, crystalline solid (1.21g, 5.7 mmol, 76%). Reaction yields vary according to the substrates and conditions used.

Mp 290-292 °C; υ_max_2825, 2551, 1673 cm^-1^; δ_H_ (400 MHz, d_6_-DMSO) 12.92 (1H, br s, O*H*), 8.01 (2H, m, C*H*Ar), 7.78 (2H, m, C*H*Ar), 7.64 (2H, m, C*H*Ar), 7.32 (2H, m, C*H*Ar), 2.37 (3H, s, C*H*_3_ ). Characterization data matches that previously reported for this compound^5^.

Survey design

Questionnaires, interviews, and focus group sessions were employed to gather student feedback, guiding the development and refinement of the ELN to align with design objectives. Following the decision to conduct a usability test, advice on survey methodology was obtained from a technology-enhanced learning expert. The survey questions were drafted and reviewed by two ELN specialists to ensure their relevance and appropriateness. A multi-item, 7-point Likert scale was used to assess seven constructs from the technology use and acceptance theory, while open-ended questions provided deeper insights into specific ELN features. After revisions, the survey was submitted to the University’s Ethics Committee for approval.

Survey Instrument

The questionnaire was administered through Microsoft Forms and distributed to students via the instructor’s email, leveraging the University's Office 365 subscription. Microsoft Forms, a free and user-friendly platform, was chosen for its accessibility across devices with internet access. Upon completion, survey responses were exported to Excel for subsequent analysis. In-person interviews were conducted, with audio recordings captured through Microsoft Teams where applicable. Open-ended survey responses and interview transcripts were imported into NVivo for thematic analysis. Coding was conducted based on the key research questions: the impact of a digital research tool on data management and technical communication in the undergraduate laboratory; whether the use of an ELN would promote the adoption of sustainable chemistry practices; and how digital interventions would influence learning and teaching in the undergraduate laboratory. Emergent codes were then grouped into recurrent themes for further analysis.

Survey Deployment

Following approval, written informed consent was obtained from students prior to survey dissemination. The pre-ELN development survey was distributed in March 2023 to gather insights into the challenges students faced with OneNote and the features they desired in an ELN. This survey targeted Year 3 and Year 4 students, with additional surveys conducted in November 2023 with a different group of Year 3 students who had not used the ELN, and in February 2024 with AI4Green project students before starting their experiments. Focus group meetings were held during lab sessions, and a post-study system usability questionnaire was administered at the end of the lab experiment.

Data Protection Declaration

Students were informed about the purpose of the survey, the data generation and processing procedures, and the potential for its use in publication through the survey notification and on the questionnaire’s landing page. No personal data was collected or stored, ensuring complete anonymity, as communicated in both the survey announcement and landing page.

Questionnaire

The questionnaires were divided into two categories: pre-app development and post-app development. The pre-app questionnaire focused on students' requirements for the ELN and challenges encountered with the existing OneNote software. The post-app questionnaire assessed ELN usage and acceptance, with sections addressing ease of use, the impact of the ELN on sustainable chemistry practices, a comparison between the ELN and OneNote, challenges faced during ELN use, and instructions for its implementation. Open-ended fields were included for additional feedback, suggestions, and improvement ideas. Some questions were designed as multi-item Likert scale questions, requiring respondents to rate their agreement on a 7-point scale. Open-ended questions were included to allow students to elaborate on their Likert scale responses. Certain questions also compared the performance of OneNote with AI4Green4Students. Cronbach’s Alpha was calculated (value=0.7) to ensure the reliability of Likert scale questions for usability testing. The Likert Scale Response Matrix used to organize the questionnaire responses is shown in **Table S1.**


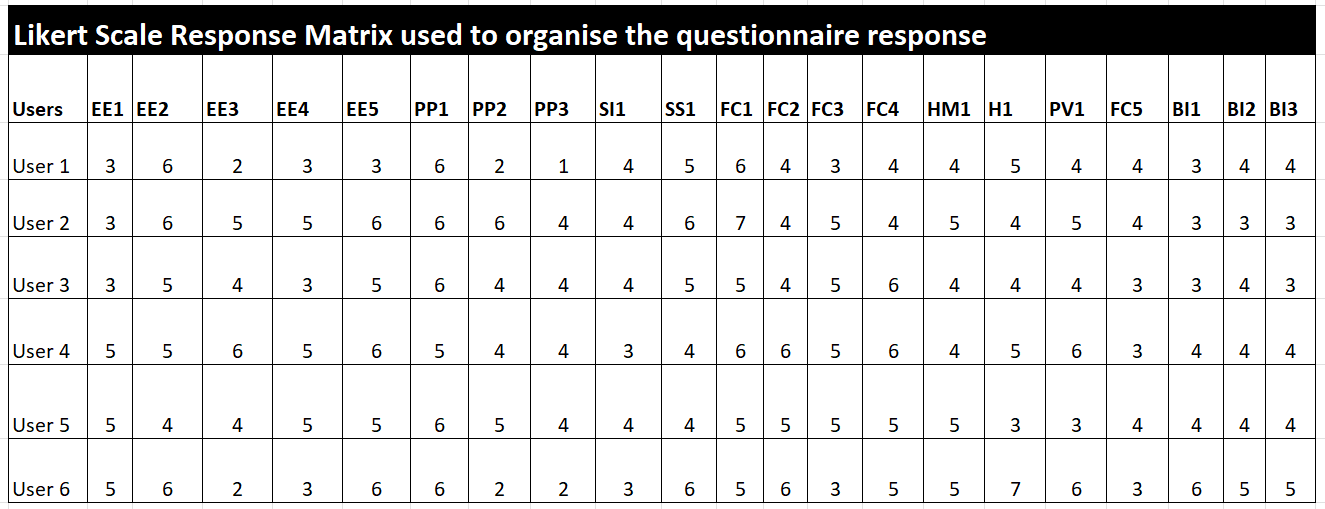


**Table S1** Likert Scale Response Matrix used to organize questionnaire responses

**Explanations**

**Rows** represent respondents; **Columns** represent each item (Item 1, Item 2, Item 3, Item 4) in the questionnaire; **Values** in the table are the respondents' answers on a Likert scale (1 = strongly disagree, 7 = strongly agree).


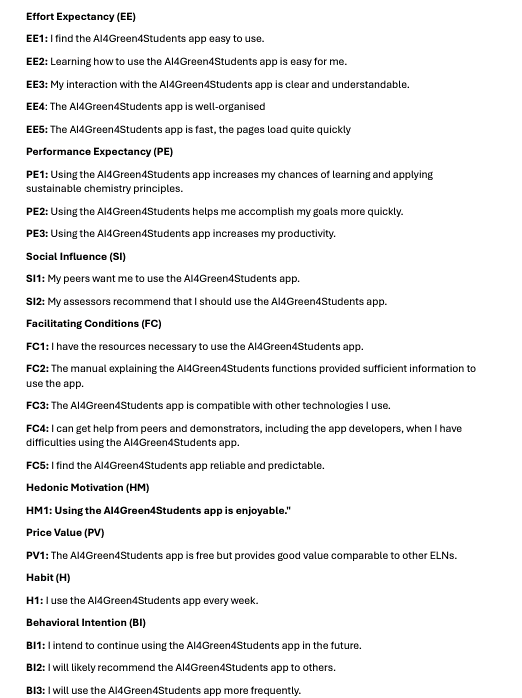


**Figure S9** Explanations of rows and columns in the Likert Scale Matrix response (Table S1)

### Pre-app deployment questionnaire for user requirements

**Section 1- Prior knowledge of green chemistry**

1. Have you studied the Sustainable Chemistry option modules in Years 1 and/or 2**?**
2. Have you considered the environmental impact of the reactions you create?
3. In what ways do you mitigate against environmental pollution in your experiments?
4. Do you think technology can help you learn sustainable chemistry?

**Section 2- Documenting and storing experimental data**

1. What methods do you use to record your notes?
2. For each of the following types of work, what do you record and how do you record it? Write the piece of information you record and check the appropriate box to show how.

|  | What you record | Paper | Electronically | N/A |
| --- | --- | --- | --- | --- |
| Doing experiments in the lab |  |  |  |  |
| Looking at literature |  |  |  |  |
| Planning your work |  |  |  |  |
| Analysing your data |  |  |  |  |
| Writing up your work |  |  |  |  |
|  |  |  |  |  |
|  |  |  |  |  |

1. When you are taking notes how do you organize them?
2. Do you use any digital notebooks? If so, which ones? E.g., OneNote, etc.
3. How easy do you find using the digital notebook?
4. What challenges do you face with the digital notebook? Please list as many as you can.
5. What improvements would you like to be made to your current digital notebook?
6. Does the notebook allow you to share data with others?
7. How easy do you share your work with others?
8. Where is your experiment data stored?
9. What could AI4Green4Students do to make your work easier?
10. Have you heard about large databases where reaction information is stored, e.g. PubChem, Reaxy and SciFinder?
11. Yes
12. No
13. Select two features you consider important to be incorporated into the AI4Green4Students app.

|  | 1st | 2nd | 3rd |
| --- | --- | --- | --- |
| Access to peer-reviewed journal article |  |  |  |
| Databases e.g. PubChem, Reaxy, etc. |  |  |  |
| Platform for planning and managing project |  |  |  |
| Machine Learning methods for data analysis and processing |  |  |  |

Post-app questionnaire to estimate the use and validity of AI4Green4Students

**AI4Green4Students app Usability Test Questionnaire**

**Section 1- Acceptance and Use of AI4Green4Students**

Please answer the following questions using a scale of 1-7:

1-Strongly Disagree; 2-Disagree; 3-Somewhat Disagree; 4-Neither Agree nor Disagree; 5-Somewhat Agree; 6- Agree; 7- Strongly Agree.

1. I find the AI4Green4Students app easy to use.
2. Using the AI4Green4Students app increases my chances of learning and applying sustainable chemistry principles.
3. Using the AI4Green4Students helps me accomplish my goals more quickly.
4. Using the AI4Green4Students app increases my productivity.
5. Learning how to use the AI4Green4Students app is easy for me.
6. My interaction with the AI4Green4Students app is clear and understandable.
7. My peers want me to use the AI4Green4Students app.
8. My assessors recommend that I should use the AI4Green4Students app.
9. I have the resources necessary to use the AI4Green4Students app.
10. The manual explaining the AI4Green4Students functions provided sufficient information to use the app.
11. The AI4Green4Students app is compatible with other technologies I use.
12. I can get help from peers, and demonstrators including the app developers when I have difficulties using the AI4Green4Students app.
13. Using the AI4Green4Students app is enjoyable.
14. The AI4Green4Students app is fast, the pages load quite quickly.
15. The AI4Green4Students app is well-organized.
16. I find the AI4Green4Students app reliable and predictable.
17. I will likely recommend the AI4Green4Students app to others.
18. I will use the AI4Green4Students app more frequently.
19. The AI4Green4Students app is free but provides good value comparable to other ELNs.
20. I use the AI4Green4Students app every week.
21. I intend to continue using the AI4Green4Students app in the future.

**Section 2 – Use and application of AI4Green4Students for Planning and Conducting Lab Experiments.**

**Please answer the following questions in detail.**

1. In how far did the app help in completing the COSHH form compared to the OneNote Notebook? If you have used anything other than OneNote Notebook please specify.
2. How did the app facilitate the planning of the experiment and writing a literature review before the actual experiment?
3. Did the learning contents, quizzes and hyperlinked websites help you in learning and applying sustainable chemistry?
4. In what way did AI4Green4Students influence your choice of solvents and catalysts in creating reactions?
5. In what way did the app improve your knowledge and understanding of how to calculate green metrics?
6. In how far would using green metrics in AI4Green4Students affect your future work?
7. How easy did you find drawing a reaction scheme using the app compared to the previously used OneNote Notebook (If your previous technology was not OneNote Notebook please specify)?
8. In how far did AI4Green4Students facilitate the recording of notes and observations?
9. How (if applicable) did AI4Green4Students influence your time management during the lab experiment?
10. What do you think about using AI4Green4Students as a method of sharing your thoughts and observations with the assessor?
11. In what ways did the feedback you received via AI4Green4Students differ from that received previously for laboratory practical experiments?
12. How has the AI4Green4Students feedback system helped in the planning and conducting of lab experiments?
13. Which of these: the AI4Green4Students app or OneNote Notebook supports you better in performing the following operations? Please enter details in the respective columns.

| **Operations** | **AI4Green4Students** | **OneNote Notebook** |
| --- | --- | --- |
| Completing prelab tasks |  |  |
| Drawing compound structures |  |  |
| Planning experiments |  |  |
| Recording experiment notes |  |  |
| Recording observations |  |  |
| Structuring and organizing notes |  |  |
| Editing recorded notes |  |  |
| Drawing tables |  |  |
| Entering data into tables |  |  |
| Sharing experiment data with members of your project group |  |  |
| Calculating yield |  |  |
| Predicting suitable catalysts |  |  |
| Analyzing data |  |  |
| Evaluating the sustainability of reactions |  |  |
| Receiving feedback from Instructors |  |  |
| Writing reports |  |  |
| Uploading files |  |  |
| Time management |  |  |

1. Are there any disadvantages to using AI4Green4Students to deliver feedback? If yes, please specify.
2. In your opinion what are the biggest advantages of using AI4Green4Students?
3. In your opinion what are the biggest disadvantages of using AI4Green4Students?

**Section 3- Application of AI4Green4Students as ELN for Data Management**

1. What are your thoughts about an electronic system for the recording of an experiment now you have carried out the practical?
2. Which features of the AI4Green4Students app were the most useful?
3. Why do you find them most useful?
4. Do you think AI4Green4Students will speed up the process of recording your experiments? Yes or No.
5. How do you think the AI4Green4Students app will affect searching for and retrieving stored chemical data?
6. How do you think the AI4Green4Students app will affect entering data in a table?
7. How has AI4Green changed the way in which you work? (E.g. the order of things, where you record different bits of information)?
8. Is there any difference in the data/observations/any other information that you have recorded using AI4Green as part of your work, compared to when you were not using it? If so please specify.
9. Do you think that using AI4Green4Students would make it the same, more or less likely for another person to recreate your experiment?

**Section 4- General Opinion**

1. Are there any experiences that stop you from accomplishing the goals of using the app?
2. Do you have suggestions on how the design and layout of the AI4Green4Students app could be improved?
3. What improvements would you like if you were to use AI4Green4Students daily?
4. Use this space for further comments, suggestions and criticisms.

# **Survey Analysis**

**Eliciting User Requirements Before the App Development**

Both student groups participating in the AI4green project and those not involved were included in this study. After the survey, user feedback was analyzed to ascertain their specific needs. A critical question investigated was how students perceived the application of green chemistry in their laboratory work; approximately 80% of the students admitted to having limited knowledge, attributing this to the lack of exposure. It was noted that although ‘sustainable chemistry' was offered as an optional module in their first and second years, many students had not opted to take it. Consequently, there was a collective suggestion from the students to integrate sustainable chemistry content directly into the app, providing them with readily accessible and essential information.

Inquiring further into the role of technology in enhancing the understanding and application of sustainable chemistry yielded unanimous agreement among the students, each affirmed that technology could indeed facilitate these aspects. Subsequent questions aimed to delve deeper into how technology aids their learning and the specific types they employ; the detailed responses to these inquiries are compiled and presented in **Table S2**.


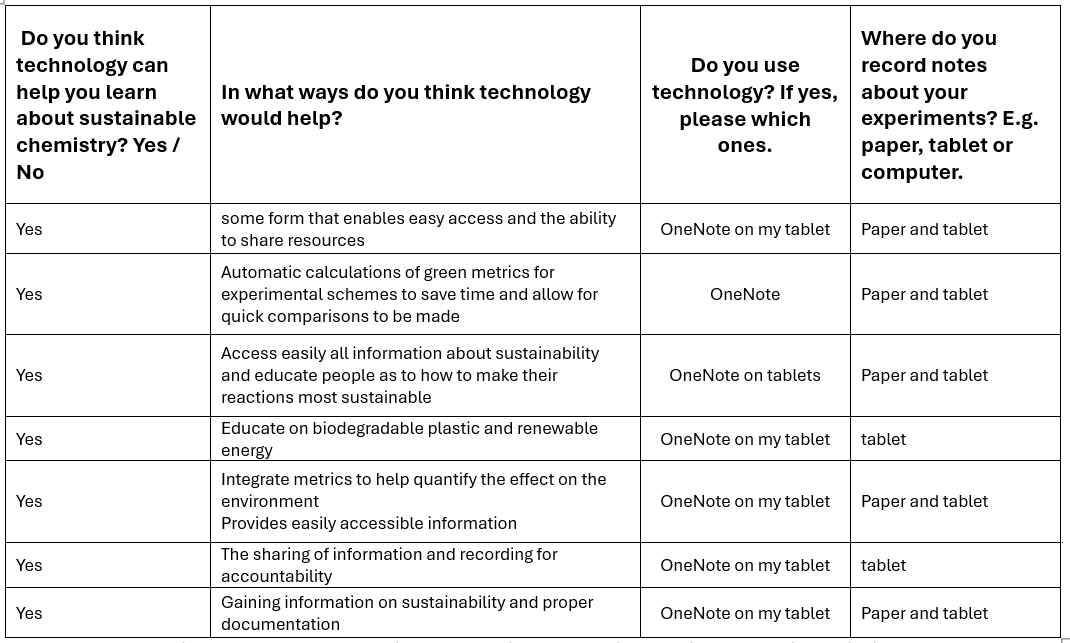


**Table S2**: The importance of technology in understanding and applying sustainable chemistry principles

From the feedback synthesized from the table above, students articulated a significant need for technology that effectively integrates the learning and application of sustainable chemistry. Easy access to information was deemed crucial to bridging knowledge gaps, especially for those who had not enrolled in elective sustainable chemistry modules. Students also expressed an acute awareness of the environmental impact of their laboratory reactions but lamented the absence of adequate tools to implement necessary changes. The discussions extended to their desire for features that would enable selections of sustainable solvents and catalysts, and encourage sustainable practices like proper documentation, data storage, and sharing skills deemed essential for their future industrial careers.

Moreover, while Table 2 indicated that all students were utilizing OneNote technology on tablets, it was surprisingly revealed that they predominantly recorded their experiment notes on paper. Probing the reasons for this apparent discrepancy, technical challenges with OneNote were uncovered. Students reported difficulties related to drawing reaction schemes, structuring tables, and logically organizing their notes within the software. Common issues included the cumbersome nature of drawing schemes which often resulted in messy and illegible outputs, challenges in inserting missed information into correct sections after completion, and operational inefficiencies like time lags in saving work, interface freezing, and complications in importing files. Consequently, students resorted to writing initial notes on paper and later transcribing these into OneNote outside of the laboratory setting, inadvertently increasing their workload and extending the time spent on notetaking, which can affect other academic responsibilities.

In an additional line of inquiry, students were asked about the specific functionalities they hoped to see implemented in the AI4Green4Students app. Contrary to popular belief that students might be reticent about sharing information, approximately 90% of respondents expressed a preference for features that facilitated sharing and collaboration (as illustrated in **Figure S10**) and 100% advocated for a structured template to facilitate data documentation, with a reaction sketcher for drawing reaction schemes. Drawing on the feedback from both students and their instructors, the AI4Green4Students app was developed, integrating features that will promote the application of sustainable chemistry and enhance data literacy and analytical capabilities.


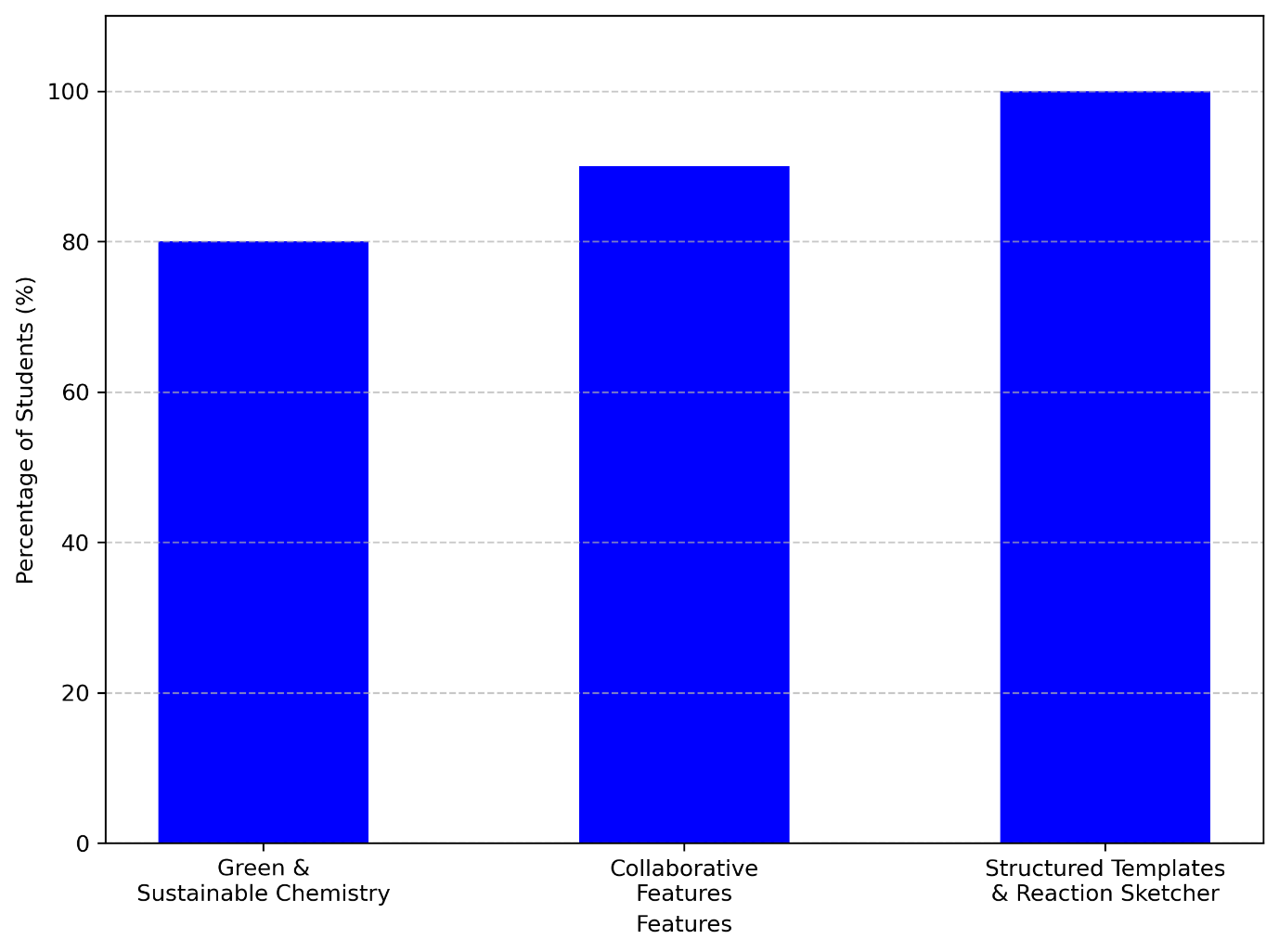


**Figure S10** Student’s proposed features of AI4Green4Students

Student’s Report – referencing metrics

Following the implementation of AI4Green4Students, discussion of sustainability metrics within student reports was observed, in contrast to previous years. This suggests a notable improvement in students' understanding of sustainability concepts. Selected excerpts from the reports are presented below.

**File name: 72% report (3)**

Page 9, line 21

“Variations in observed metrics due to base concentration can also be seen, increase to concentration appears to have a positive effect on yield and E-factor. In reaction 11 use of 3M sodium carbonate results in one of the highest recorded RMEs, of 39.4%”

Page 9, line 6

“Due to the larger toxicity of THF over water……, water will be used for continued experiments”

**File name: 71% report**

Page 12, line 13

“There appeared to be a correlation in using a higher concentration and/or a lower equivalence [sic] of base whereby the latter saw deeply improved metrics (Table 1) and the former allowed for a reduced total input quantity whilst maintaining acceptable yields.”

Page 8, line 29

“Furthermore, the increased yield, and evidently improved greenness by lowering input observed under 1 equivalent of base against 2 and 4 equivalents (ca.10% yield, 20 EF and 5% RMI improvement) allows the optimal yield and base quantity for greenness”

Page 8, line 33

“combining this with the increased base concentration to 3M (which resulted in steady 75-80% yields) could enhance the metrics observed even further due to its reduced total input and prosperous yields.”

**File name: 69% report**

Page 4, line 17

“Although lower temperatures such as 20 °C and 50 °C reduce energy consumption, they were not sustainably viable as the e-factor [sic] and RME were relatively poor.”

**File name: 69% report (4)**

Page 5, paragraph 3

“the amount of base being reduced gave good yields this means that using 3eq might not be necessary and we can use less base this is essential as it reduces the e-factor of the reaction [whilst] retaining the amount of product [sic].”

Page 6, “Conclusion”

“Overall it was seen that lower amounts of base and higher concentrations of base worked well for this reaction to be able to reduce waste.”

Sustainability Cheat Sheet

This overview outlines the United Nations' sustainability development goals, and the role chemistry plays in achieving them. Students who did not choose the sustainability module in year 1 and year 2 will find this reference guide helpful in creating sustainable reactions.

**What is sustainability?** The three pillars of sustainability are Environment, Society and Economy.


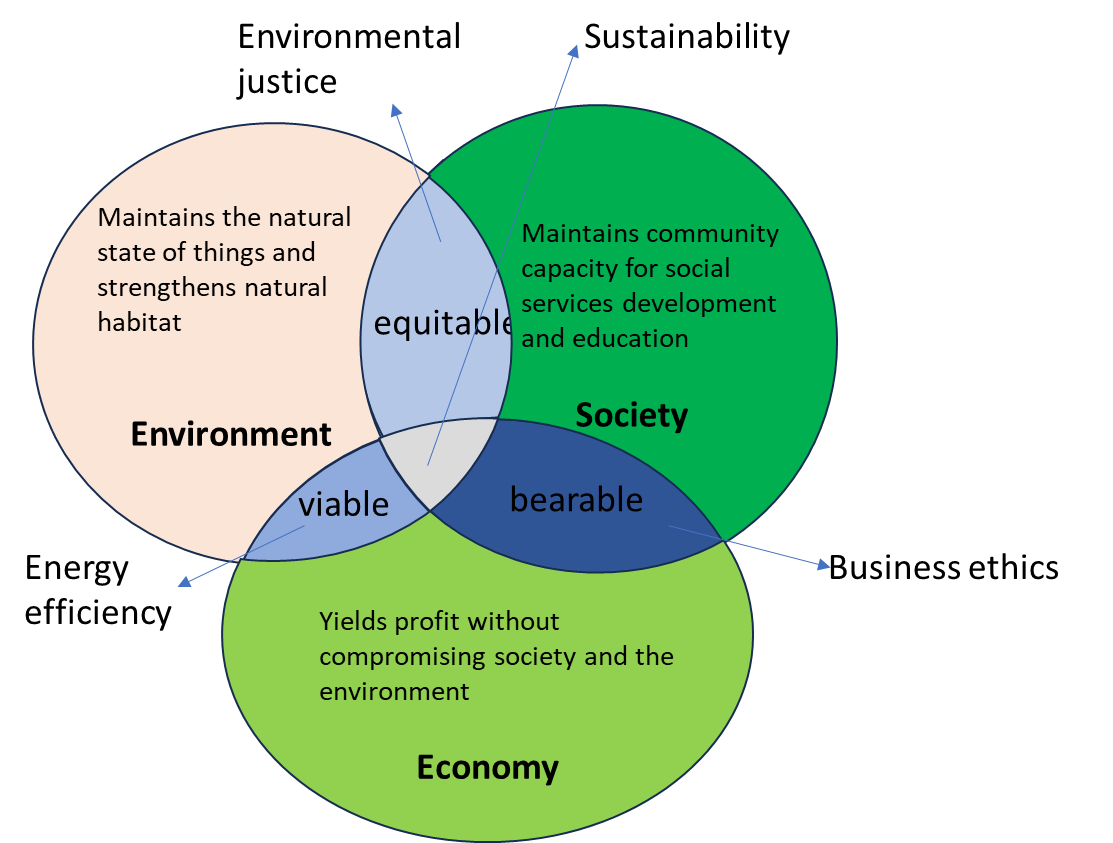


**Figure S11** Venn diagram showing the relationship between the three pillars of sustainability.

The United Nations (UN) created guidelines that will help organisations and businesses evaluate the sustainability of their activities^6^. Figure S12 shows these UN sustainability development goals (UNSDGs).

**Figure S12** The United Nations Sustainability Development Goals.

**How can chemistry contribute to achieving the UNSDGs?** This can be achieved via the incorporation of green and sustainable chemistry into chemistry practice to create reactions and products that are safe for humans and have minimal impact on the environment.

**What is sustainable chemistry?** It is the chemistry that uses resources, including energy, at a rate at which they can be replaced naturally, and the generation of waste cannot be faster than the rate of their remediation^1^. It comprises both the impressions of green chemistry and the effects of processing, materials, energy, and economics. Sustainable chemistry lies at the intersection of the Venn diagram marked sustainability. It meets the needs of the three pillars.

**What is green chemistry?** It is the design of chemical products and processes that reduce or eliminate the use or generation of hazardous substances. It provides the methodology for changing the intrinsic nature of a chemical product or process so that it is of less risk to humans and the environment. The 12 principles of green chemistry^7,8^ are a framework for learning about green chemistry and designing greener reactions (Table S3).

| **Principle** | **Meaning** |
| --- | --- |
| 1 | It is better to prevent waste than to treat or clean up waste after it has been created. |
| 2 | Design synthetic methods to incorporate all raw materials into the final product. |
| 3 | Design synthetic methods that generate substances with little or no toxic effect on humans and the environment |
| 4 | Design chemical products to preserve the efficacy of function while reducing toxicity. |
| 5 | Avoid the use of auxiliary solvents, but if used must be benign |
| 6 | Design for energy efficiency. Synthetic methods should be conducted at ambient temperature. |
| 7 | Minimise the use of non-renewable feedstock and use renewable ones. |
| 8 | Avoid or minimise unnecessary derivatizations because they require additional reagents and can generate waste. |
| 9 | Use catalytic reagents instead of inorganic stoichiometric reagents |
| 10 | Design chemical products that will degrade into innocuous products after use. |
| 11 | Design analytical methodologies that allow for real-time, in-process monitoring and control for pollution prevention. |
| 12 | Use substances that will minimise the potential chemical accidents in a chemical process. |

**Table S3** The 12 Principles of Green Chemistry.

**What are green metrics?** These are parameters used to evaluate the greenness of a reaction quantitatively^9,10^. Some examples of green metrics are shown in Table S4.

| **Metrics** | **Expression** |
| --- | --- |
| E factor | Total waste(kg)/products(kg) |
| Atom Economy (AE) | (FW product/FW of all reactants used in reaction) X 100 |
| Reaction mass efficiency (RME) | (Mass of isolated product/total mass of reactants used in reaction) X100 |
| Carbon efficiency (CE) | (Mass of carbon in product/total mass of carbon in reactants) X100 |
| Process mass intensity (PMI) | Total mass in a process or process step/mass of product |

**Table S4** Some of the Green metrics for evaluating the ‘greenness’ of a reaction.

1. **E factor**- measures the quantity of waste that is produced for a given mass of product.

2. **Atom Economy**- calculates how much of the reactants remain in the final product.

3. **Reaction mass efficiency**- measures the efficiency with which reactants are converted into the desired product.

4. **Carbon efficiency**- measures the percentage of carbon in the reactants that remain in the final product. This leads to the potential generation of greenhouse gases.

5. **Process mass intensity**- measures the total mass input in a process or process step relative to the final product.

6. **Solvent metrics-** evaluates the toxicity and environmental impact of solvents used in a chemical reaction process or process step. A separate document showing Sanofi and GlaxoSmithKline guides for selecting solvents is included for your use.

**References**

(1) Kanza, S.; Willoughby, C.; Knight, N. J.; Bird, C. L.; Frey, J. G.; Coles, S. J. Digital Research Environments: A Requirements Analysis. *Digit. Discov.* **2023**, *2* (3), 602–617. https://doi.org/10.1039/D2DD00121G.

(2) Kanza, S.; Willoughby, C.; Gibbins, N.; Whitby, R.; Frey, J. G.; Erjavec, J.; Zupančič, K.; Hren, M.; Kovač, K. Electronic Lab Notebooks: Can They Replace Paper? *J. Cheminformatics* **2017**, *9* (1), 31. https://doi.org/10.1186/s13321-017-0221-3.

(3) Nizma, C.; Bangun, R.; Benhur, B.; Cahyoginarti, C.; Zuardi, M. The Role of Organizational Structure in Project Management. *J. Syntax Transform.* **2024**, *5*, 590–597. https://doi.org/10.46799/jst.v5i2.928.

(4) Hamilton, A. E.; Buxton, A. M.; Peeples, C. J.; Chalker, J. M. An Operationally Simple Aqueous Suzuki–Miyaura Cross-Coupling Reaction for an Undergraduate Organic Chemistry Laboratory. *J. Chem. Educ.* **2013**, *90* (11), 1509–1513. https://doi.org/10.1021/ed4002333.

(5) Yuan, M.; Song, Z.; Badir, S. O.; Molander, G. A.; Gutierrez, O. On the Nature of C(Sp3)-C(Sp2) Bond Formation in Nickel-Catalyzed Tertiary Radical Cross-Couplings: A Case Study of Ni/Photoredox Catalytic Cross-Coupling of Alkyl Radicals and Aryl Halides. *J. Am. Chem. Soc.* **2020**, *142* (15), 7225–7234. https://doi.org/10.1021/jacs.0c02355.

(6) *THE 17 GOALS | Sustainable Development*. https://sdgs.un.org/goals (accessed 2024-05-29).

(7) Green, S. A. Green Chemistry: Progress and Barriers. *Phys. Sci. Rev.* **2016**, *1* (10), 20160072. https://doi.org/10.1515/psr-2016-0072.

(8) Anastas, Paul T; John C Warner. *Green Chemistry: Theory and Practice*, online edition.; Oxford Academic: Oxford, 2000.

(9) Jiménez-González, C.; Constable, D. J. C.; Ponder, C. S. Evaluating the “Greenness” of Chemical Processes and Products in the Pharmaceutical Industry—a Green Metrics Primer. *Chem Soc Rev* **2012**, *41* (4), 1485–1498. https://doi.org/10.1039/C1CS15215G.

(10) Constable, D. J. C.; Curzons, A. D.; Cunningham, V. L. Metrics to ‘Green’ Chemistry—Which Are the Best? *Green Chem* **2002**, *4* (6), 521–527. https://doi.org/10.1039/B206169B.
