# Supplementary material for: AI4Green4Students: Promoting Sustainable Chemistry in Undergraduate Laboratories with an Electronic Lab Notebook
Source: J Chem Educ. 2025 Jun 5;102(7):2720–31. doi: 10.1021/acs.jchemed.4c01393 (PMC12243079; doi:10.1021/acs.jchemed.4c01393)
Supplement: Supplementary file 3 [file ed4c01393_si_003.pdf]

Supporting Information- ELN design and evaluation

AI4Green4Students: Promoting sustainable chemistry in undergraduate laboratories with an electronic lab notebook

Peace C Nwafor [a], Shason Gurung [b], Philip van Krimpen [b], Lenka Schnaubert [c], Katherine Jolley [a], Samantha Pearman-Kanza [d], Cerys Willoughby [d], Jonathan D. Hirst [a*]

[a] School of Chemistry, University of Nottingham, University Park, Nottingham, NG7 2RD, United Kingdom

[b] Digital Research Service, University of Nottingham, University Park Nottingham, NG7 2RD, United Kingdom

[c] Learning Sciences Research Institute, School of Education, University of Nottingham, Dearing Building (C85), Jubilee Campus, Nottingham, NG8 1BB, United Kingdom

[d] School of Chemistry and Chemical Engineering, University of Southampton, University Road Southampton SO17 1BJ, United Kingdom

* Email: jonathan.hirst@nottingham.ac.uk

AI4Green4Students

Accessing AI4Green4Students

Web Browser
We recommend using Google Chrome.

URL
Type in 'ai4g4s.app' into the address bar.

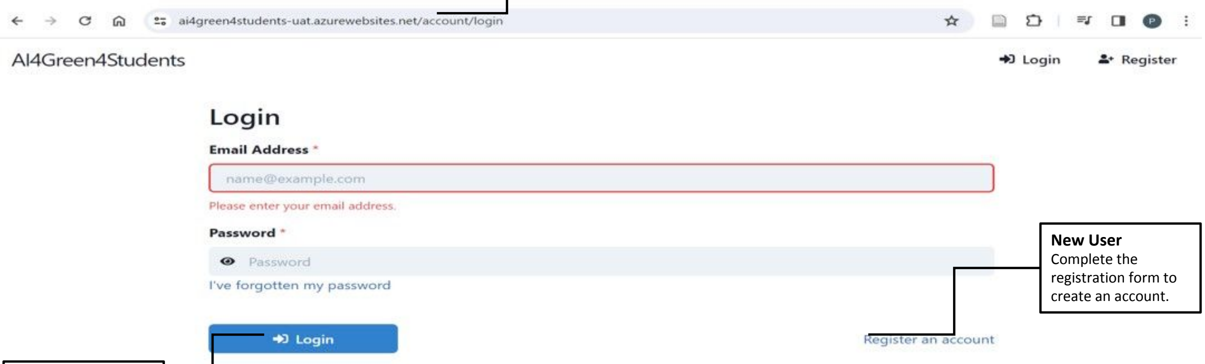

New User
Complete the registration form to create an account.

Existing User
Sign in using your login credentials.

AI4Green4Students

AI4Green4Students

Getting Started – Instructor

1 Create Project Group
This is the create projectgroup page.

If you are an instructor, you will be able to create a projectgroup by using the form on this page.

Once created you can invite registered students to join or add them manually to the group.

To invite students, use the "New" button on the user management page. Enter the email address, select a role and click invite.

You will receive an email when a student has joined the group.

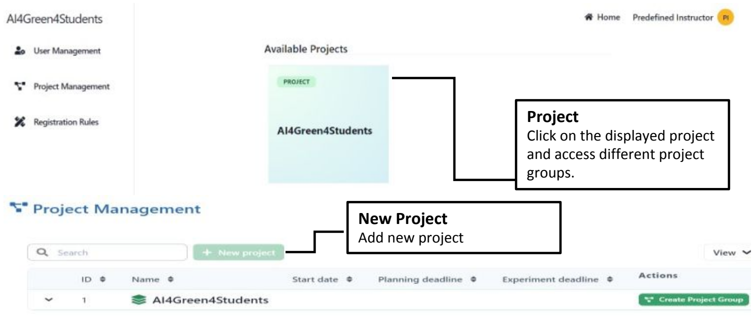

Project
Click on the displayed project and access different project groups.

New Project
Add new project

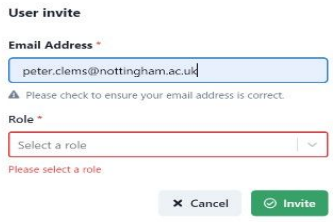

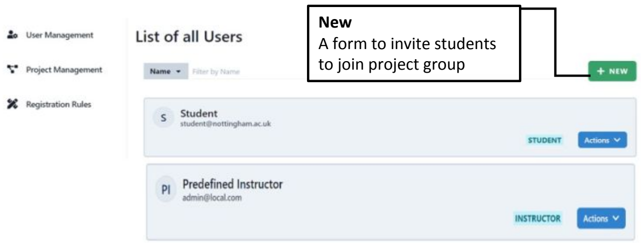

New
A form to invite students to join project group

AI4Green4Students

AI4Green4Students

To evaluate students' work – Instructor

Providing Feedback

Once an instructor has selected a project group, the list of all the students in the group will be displayed alongside the activities they have completed.

The instructor can use the action pane to view the plan and provide feedback or request for a change.

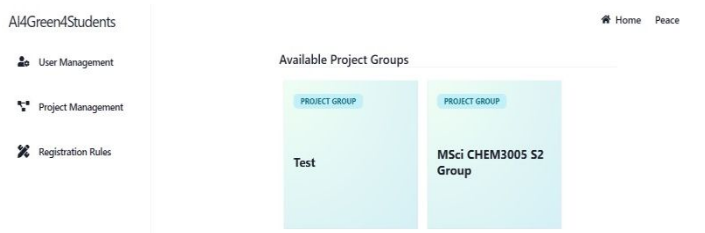

Project Group Activities
Check the group plan, showing a week-by-week plan on how to complete a project.

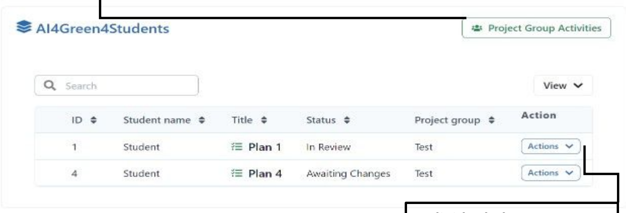

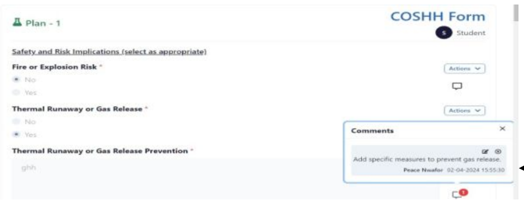

Individual Plan
Evaluate each student's plan and approve or add feedback.

AI4Green4Students

AI4Green4Students

Getting Started – Student

Learn more
Access the sustainability page. You don't need to be a member of a project group.

Sustainability metrics
Practise calculating green metrics.

Home
This is the home page

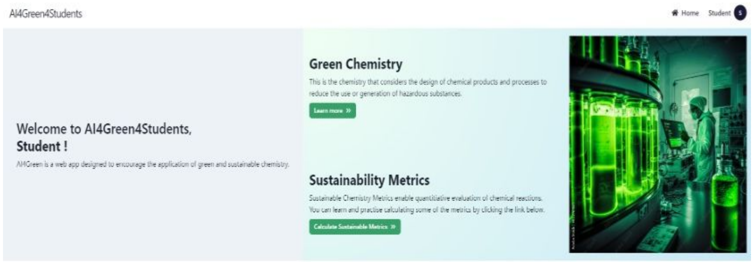

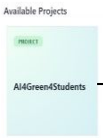

Available Projects
Select your project and project group

AI4Green4Students

AI4Green4Students

Project Group

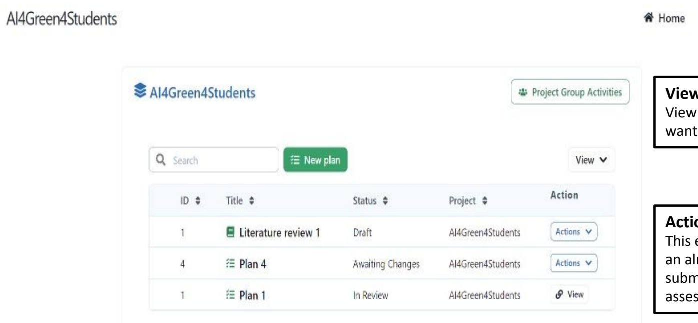

Project Activities
This encourages collaboration. Members of a project group plan the project timeline showcasing the weekly plan.

New Plan
Create a new plan to be approved before conducting experiment in the lab

Search
Search for an existing plan to edit before submission.

View
View and select the columns you want to visualise.

Action
This enables you to either view an already created plan or to submit it to the supervisor for assessment.

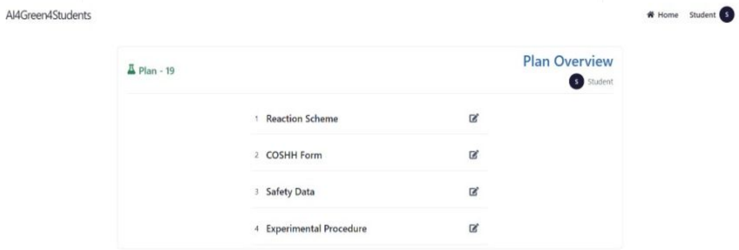

Plan Overview
Displays elements of prelab tasks to be completed. Use a Reaction scheme to draw structures and generate a reaction table.

You can use the COSSH form to assess the health and safety issues of the reaction to be created.

Help
Access our help guides and video tutorials.

AI4Green4Students

AI4Green4Students

Reactions

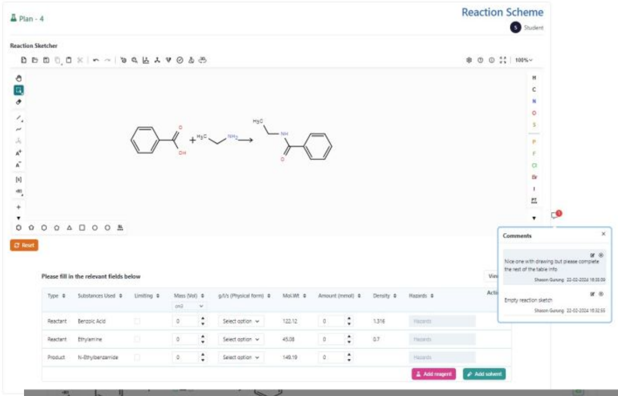

1 Sketcher
Draw the reaction here. Compounds drawn over the arrow will be ignored.
Press “Generate reaction data” to continue.

Comment
The instructor adds feedback here to aid in rework or improvements.

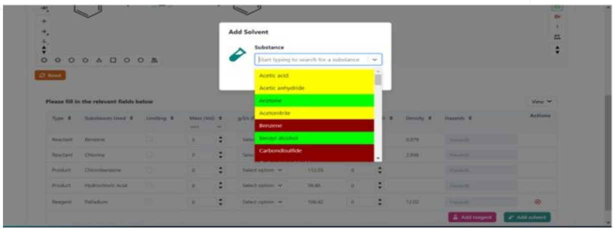

2 Reaction Table
Fill in all highlighted boxes. Add any reagents or solvents by CAS or name.

Enter the correct hazard codes. The system will validate your entry to enable you provide correct codes. Your supervisor will later assess the codes and give you feedback.
Press “Save” to proceed.

Add Solvent
Press add solvent and type into the substance box. A drop-down list of solvents colour-coded in red, green, yellow and dark red will be displayed. This enables the selection of greener solvents.

AI4Green4Students
