# Supplementary material for: AI4Green4Students: Promoting Sustainable Chemistry in Undergraduate Laboratories with an Electronic Lab Notebook
Source: J Chem Educ. 2025 Jun 5;102(7):2720–31. doi: 10.1021/acs.jchemed.4c01393 (PMC12243079; doi:10.1021/acs.jchemed.4c01393)

Supporting Information- ELN design and evaluation

**AI4Green4Students: Promoting sustainable chemistry in undergraduate laboratories with an electronic lab notebook**

Peace C Nwafor ^a^, Shason Gurung ^b^, Philip van Krimpen ^b^, Lenka Schnaubert ^c^, Katherine Jolley ^a^, Samantha Pearman-Kanza ^d^, Cerys Willoughby ^d^, Jonathan D. Hirst ^a*^

^a^ School of Chemistry, University of Nottingham, University Park, Nottingham, NG7 2RD, United Kingdom

^b^ Digital Research Service, University of Nottingham, University Park Nottingham, NG7 2RD, United Kingdom

^c^ Learning Sciences Research Institute, School of Education, University of Nottingham, Dearing Building (C85), Jubilee Campus, Nottingham, NG8 1BB, United Kingdom

^d^ School of Chemistry and Chemical Engineering, University of Southampton, University Road Southampton SO17 1BJ, United Kingdom

* Email: jonathan.hirst@nottingham.ac.uk


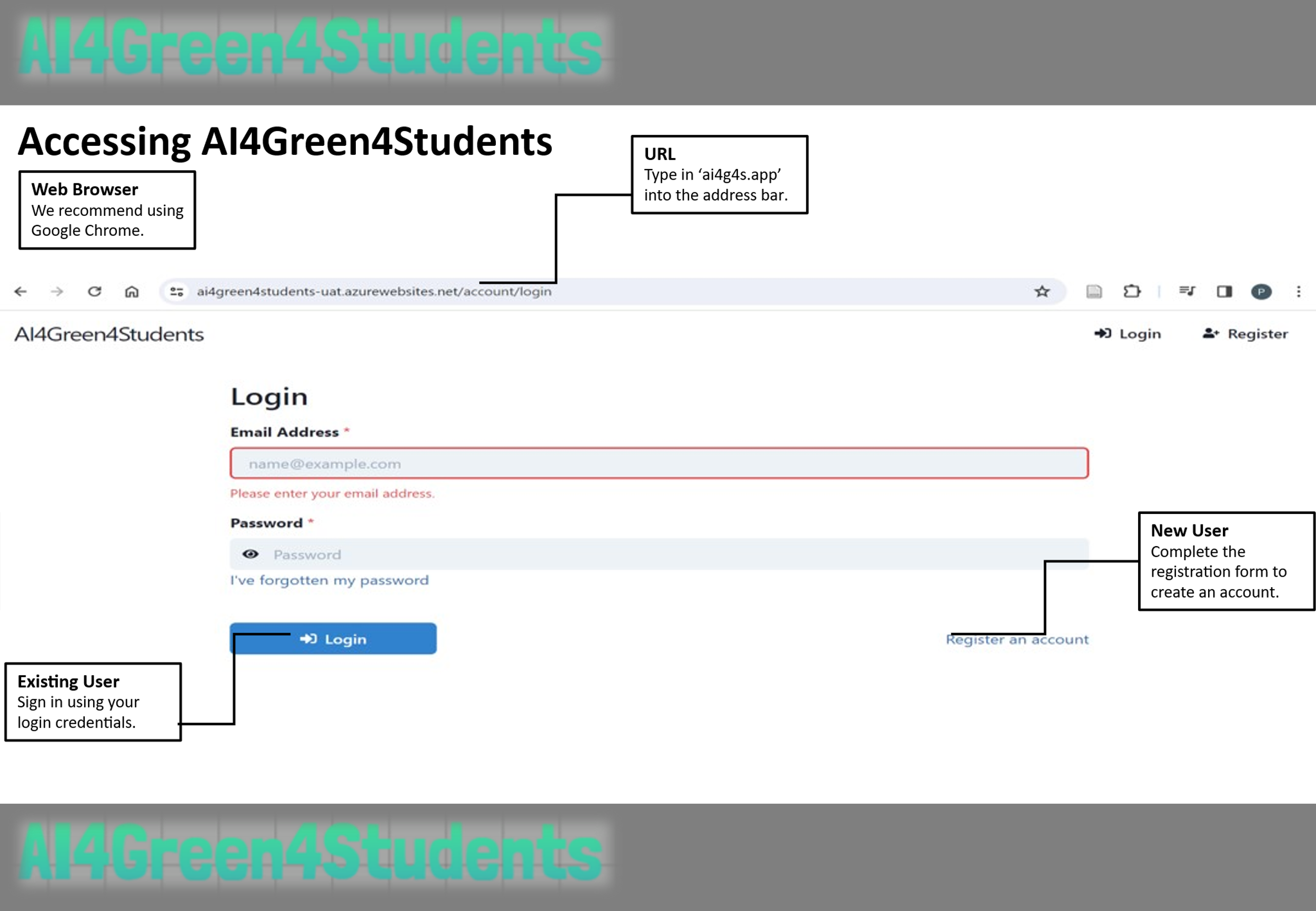


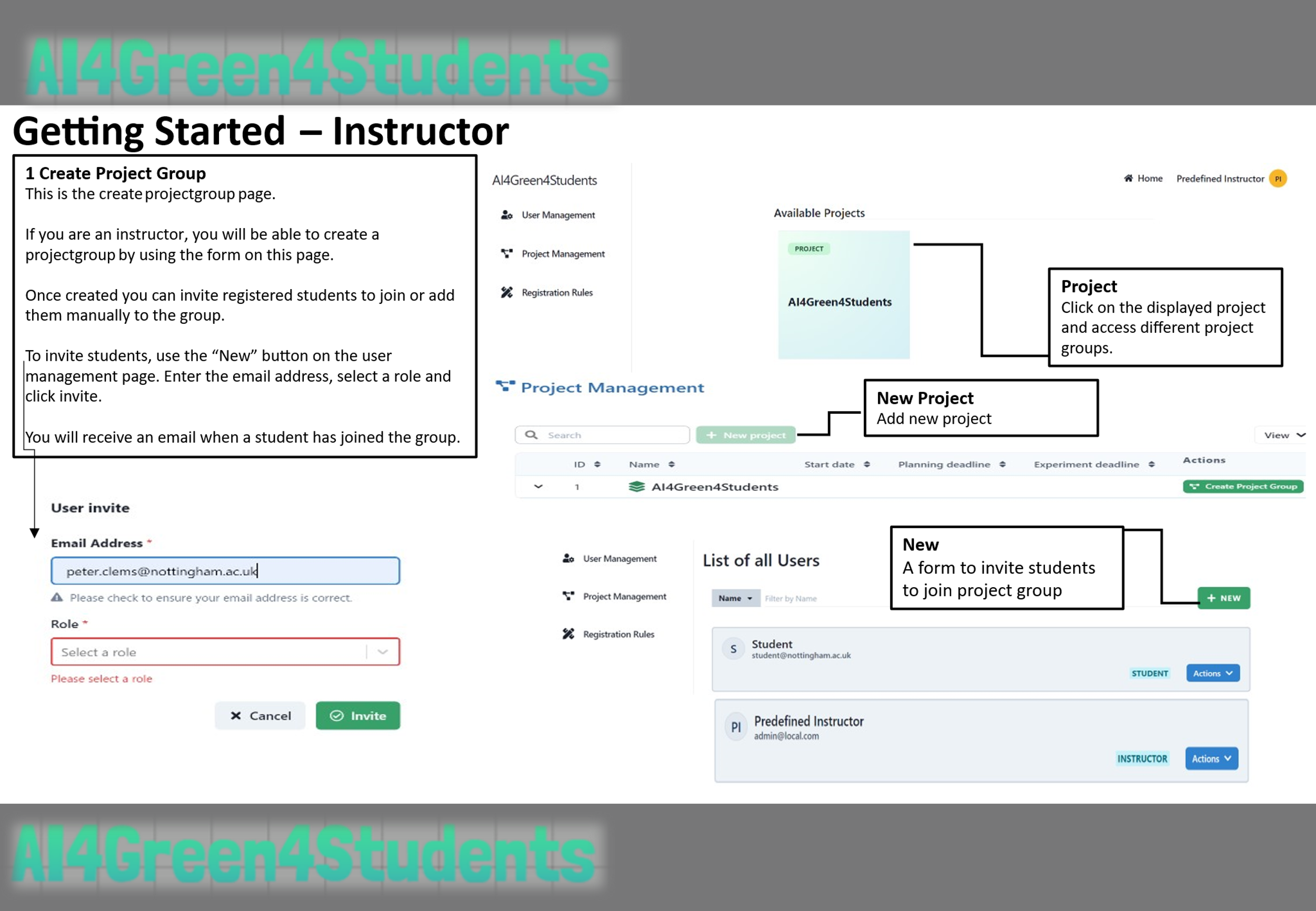


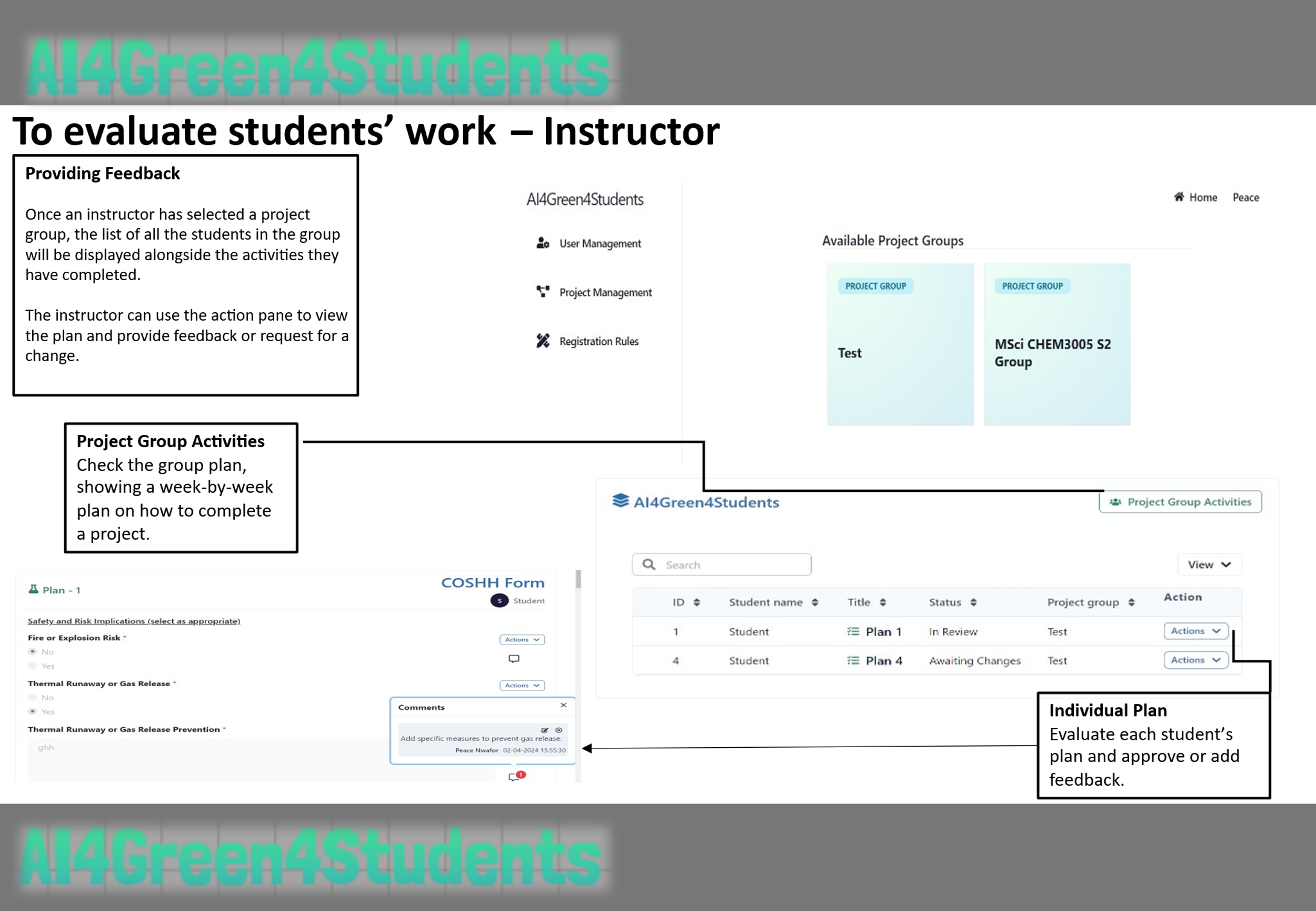


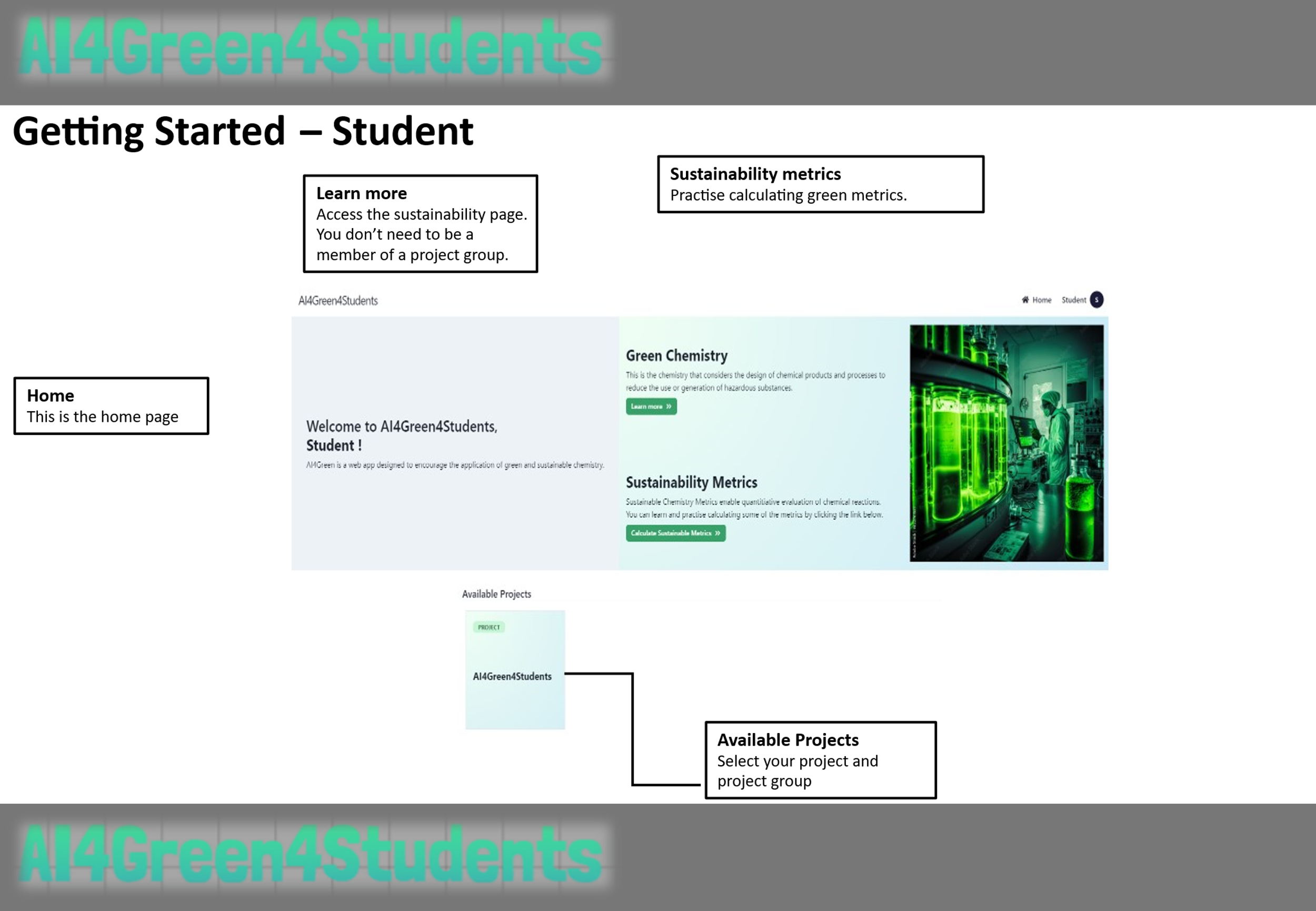


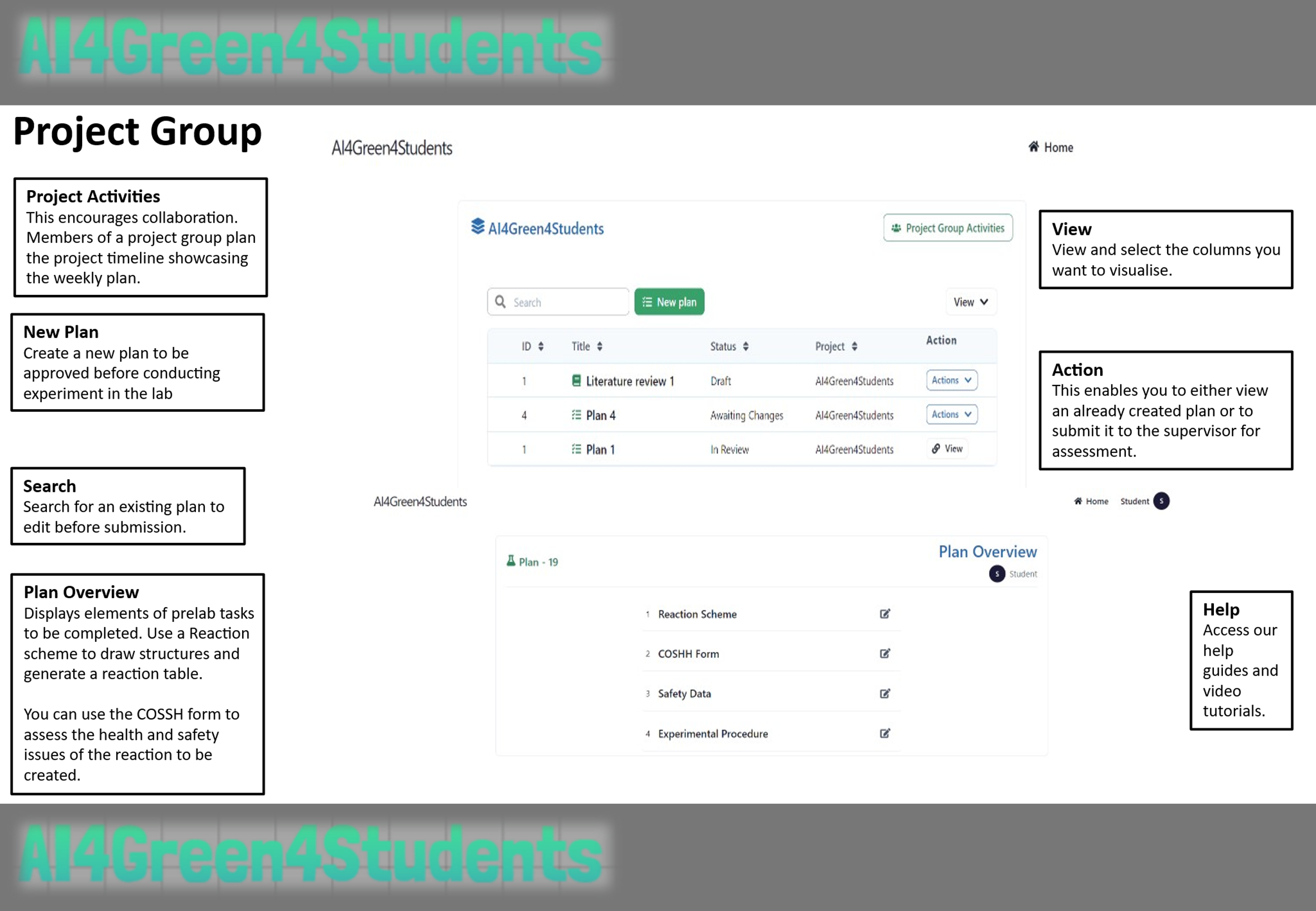


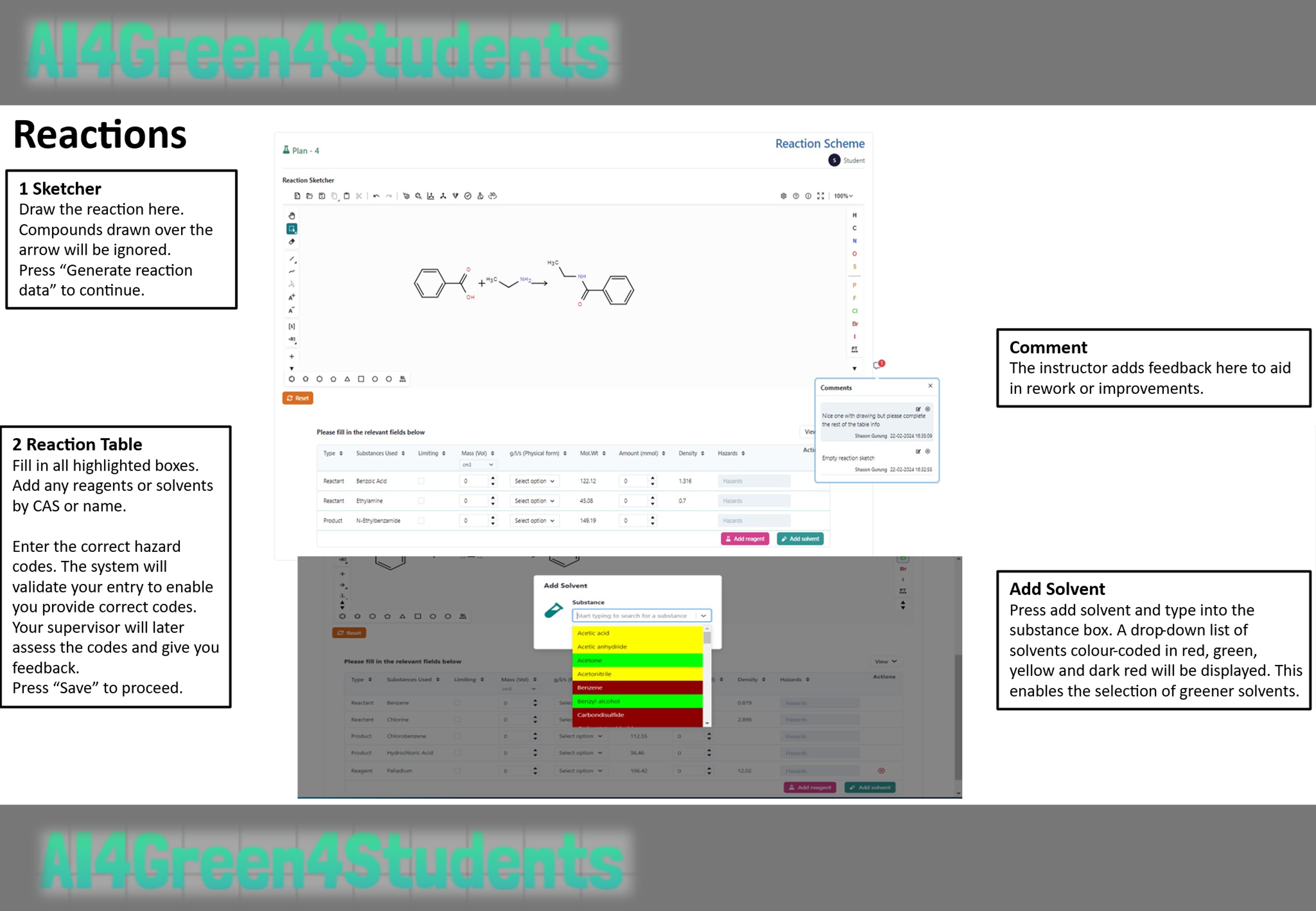

Supplement: Supplementary file 4 [file ed4c01393_si_004.docx]
